# Signaling cascades transmit information downstream and upstream but unlikely simultaneously

**DOI:** 10.1186/s12918-016-0303-2

**Published:** 2016-08-25

**Authors:** Simona Catozzi, Juan Pablo Di-Bella, Alejandra C. Ventura, Jacques-Alexandre Sepulchre

**Affiliations:** 1 Université Côte d’Azur, CNRS, INLN, 1361 route des lucioles, Valbonne, 06560 France; 2 IFIBYNE-UBA-CONICET and Departamento de Fisiología, Biología Molecular y Celular, Facultad de Ciencias Exactas y Naturales, Universidad de Buenos Aires, Ciudad Universitaria, Pabellón II, Buenos Aires, C1428EHA Argentina

**Keywords:** Signaling cascades, Retroactivity, MAPK cascades, Drug design, Kinase inhibitors

## Abstract

**Background:**

Signal transduction is the process through which cells communicate with the external environment, interpret stimuli and respond to them. This mechanism is controlled by signaling cascades, which play the role of intracellular transmitter, being able to transmit biochemical information between cell membrane and nucleus. In theory as well as in practice, it has been shown that a perturbation can propagate upstream (and not only downstream) a cascade, by a mechanism known as retroactivity. This study aims to compare the conditions on biochemical parameters which favor one or the other direction of signaling in such a cascade.

**Results:**

From a mathematical point of view, we show that the steady states of a cascade of arbitrary length *n* are described by an iterative map of second order, meaning that the cascade tiers are actually coupled three-by-three. We study the influence of the biochemical parameters in the control of the direction of transmission – upstream and/or downstream – along a signaling cascade. A numerical and statistical approach, based on the random scan of parameters describing a 3-tier signaling cascade, provides complementary findings to the analytical study. In particular, computing the likelihood of parameters with respect to various *signaling regimes*, we identify conditions on biochemical parameters which enhance a specific direction of propagation corresponding to forward or retro-signaling regimes. A compact graphical representation is designed to relay the gist of these conditions.

**Conclusions:**

The values of biochemical parameters such as kinetic rates, Michaelis-Menten constants, total concentrations of kinases and of phosphatases, determine the propensity of a cascade to favor or impede downstream or upstream signal transmission. We found that generally there is an opposition between parameter sets favoring forward and retro-signaling regimes. Therefore, on one hand our study supports the idea that in most cases, retroactive effects can be neglected when a cascade which is efficient in forward signaling, is perturbed by an external ligand inhibiting the activation at some tier of the cascade. This result is relevant for therapeutic methodologies based on kinase inhibition. On the other hand, our study highlights a less-known part of the parameter space where, although the forward signaling is inefficient, the cascade can interestingly act as a retro-signaling device.

**Electronic supplementary material:**

The online version of this article (doi:10.1186/s12918-016-0303-2) contains supplementary material, which is available to authorized users.

## Background

Cell signaling is responsible for the development and functioning of both unicellular and multicellular organisms. Abnormal cell signaling leads to diseases which involve at least one breakdown in cell communication [1].

Signaling pathways control and regulate the flow of biochemical information between cells and their external environment, which is essential for cell signaling. Typically, a stimulus (in most cases molecules secreted by another cell, e.g. growth factors, hormones) is detected on the surface of the plasma membrane, activating complex signaling. Covalent modification cycles are one of the major intracellular signaling mechanisms, both in prokaryotic and eukaryotic organisms [2]. Kinase cascades are a sequence of such cycles, in which the activated protein in one tier promotes the activation of the protein in the next one. The advantages of these cascades in signal transduction are multiple and the conservation of their basic structure throughout evolution suggests their usefulness. A reaction cascade may amplify a weak signal, accelerate the speed of signaling, steepen the profile of a graded input as it propagates, filter out noise in signal reception, introduce time delay, and allow alternative entry points for differential regulation [3–5].

**Fig. 1:**
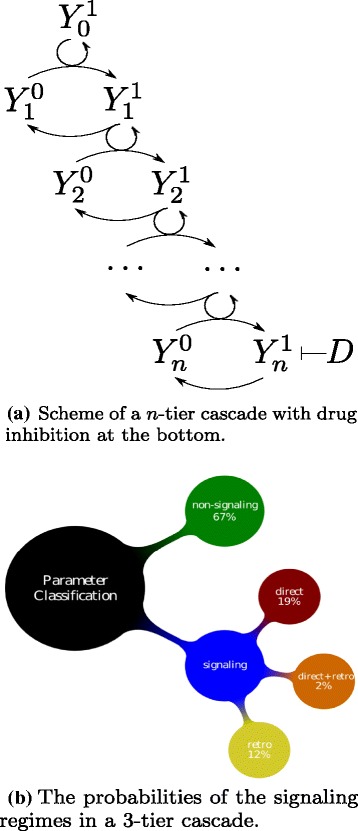
A linear cascade propagates signals in different directions with a certain probability

Recent theoretical and experimental studies have demonstrated that kinase cascades exhibit bidirectional signal propagation via a phenomenon termed retroactivity [6–11]. This phenomenon arises because cycles in a cascade are coupled with both the next and the previous cycle (Fig. 1a). The cycles can be thought of as modules, where each module’s substrate sequesters a key component of the previous one, limiting the component’s ability to participate in the previous module and inducing a natural change on it. This change may then propagate upstream through one or more preceding modules.

In [6, 11–14] the effect of retroactivity in kinase cascades has been investigated. Applying a perturbation at any level of the cascade (such as sequestration of the active protein or over-expression of a phosphatase) would have implications both downstream and upstream of the disturbance level due to retroactivity. This result, that was experimentally validated ([10, 15–17]), indicates that a kinase cascade is a bidirectional device regarding information transmission. However, how likely is it that a cascade transmits information upstream? If parameter conditions favor this last situation, can this coexist with standard signal propagation down the cascade? Some of the results in [6, 11–14] show evidences that favorable conditions to forward signaling are typically opposite to conditions promoting retroactive signaling.

In [6], an arbitrary long cascade (with every unit in steady-state) has been considered to be locally perturbed and two different regimes have been identified (see Fig. 4 therein). The first regime (perturbation traveling mostly downstream) is achieved when both all kinases and all phosphatases are saturated by their substrates, making the amount of protein increase down the cascade. The opposite regime is attained when only the phosphatases are saturated. These two regimes involved the relaxation of a perturbation, and was actually a first evidence that separated regions in the parameter space of a cascade might characterize the propagatation of a signal downstream or upstream.

In [11] it has been explored how a small perturbation in the concentration of an inhibitor of the active protein at the last level perturbs the steady-state concentrations of a relatively long linear the cascade. It has been recognized that natural cascades can amplify a perturbation (for free active protein) as it propagates upstream, but the probability of attenuation is substantially higher than that of amplification. In addition, the probability of attenuation increases with the number of stages in the cascade. Interestingly, the parameter conditions that produce an attenuation of the upstream response ensure the amplification of downstream signaling.

In [12] the authors have focused on kinase inhibitors, a class of targeted therapies designed to interfere with a specific kinase molecule in a dysregulated signaling pathway. Within physiologically and therapeutically relevant ranges for all parameters, a targeting inhibitor can naturally induce an off-target effect via retroactivity, having the capacity of turning “on" an otherwise “off" parallel cascade when two cascades share an upstream activator. In that study it was mainly considered a network of three covalent modification cycles: an upstream cycle (cycle 1) activating two parallel cycles. A perturbation was applied to one of the downstream cycles (cycle 3) and the effect measured in the other one (cycle 2); this effect reaches the upper cycle via retroactivity and then is transmitted to the parallel pathway. An optimization procedure was performed to identify ranges of the parameter values that ensure a measurable effect in cycle 2. This optimization implied the combination of a good upstream transmission of information (from cycle 3 where the perturbation is applied, to cycle 1) and a good downstream signaling (from cycle 1 to cycle 2) and noticeably, the parameter ranges characterizing these two directions of signaling were not only different but somehow opposite.

Similar conclusions have been experimentally observed on a two-branch MAP-Kinase cascade, allowing to activate responses of JNK and p38MAPK (equivalent to cycles 2 and 3 in the previous description) [17]. Here the authors termed the notion of retroactive signaling by retrograde propagation. They experimentally showed that retrograde propagation from JNK to p38MAPK is significantly higher than from p38MPAK to JNK. A preexisting theoretical study [13], enables to interpret such asymmetry by the fact that in this branched pathway, one side is more effective than the other for forward signaling whereas the second is more effective for retroactive signaling. In particular, for the simplest case of a bicyclic kinase cascade, that study has analyzed the conditions for which the upstream cycle was affected: either by a change of the total amount of protein in the downstream cycle, or by a variation of the phosphatase deactivating the same protein. Notably, it was revealed that when the downstream cycle was mostly deactivated, thus impeding a forward signaling, the retroactive effects on the upstream cycle were larger.

In this paper, we address the question of simultaneous bidirectionality in signaling cascades, and we use both analytical and numerical approaches. Our main goal is to develop a comparative study of parameters affecting forward or retroactive responses in linear signaling cascades (Fig. 1a). In the first part we develop an analytical study of the dose-response curve, defined as the concentration of the last activated protein in the cascade as a function of the initial stimulus. We also consider that a drug can be added in the cell in order to inhibit the last activated protein in the cascade. Our aim being to examine the features of the dose-response as a function obtained from a discrete iterative map with boundary conditions, and thus optimize the forward signaling. Moreover, we define the drug-response curves, as the concentration of the intermediate activated proteins as a function of the inhibiting drug, which refer to the retroactive (backward) signaling. In the second part, we perform a numerical investigation on a 3-tier cascade in order to test the cascade for uni- and bidirectional propagation (upstream and/or downstream) along it. The cascade parameters are sampled and classified according to the signaling direction they contribute to. Some striking results are already summarized on Fig. 1b as follows: 67 % of parameters lead to no form of signaling; 19 % of parameters show forward signaling without retroactivity; 12 % of parameters enable retroactive response but no forward signaling; 2 % of parameters show both forward and retroactive signaling properties.

Evidently, any estimate of such probabilities strongly depends on the assumed distribution of biochemical parameters from which the sampling is performed. Presumably, the actual parameter distribution existing in natural signaling pathways is not uniform. On the other hand, this knowledge is currently out of reach, or would be very hard to access. Therefore in this paper we choose as a reference point, a *uniform* distribution of biochemical parameters lying in some predefinite ranges. However, the fact that numerous efficient signaling cascades, and retroactivity effects, have been measured experimentally, suggests that the estimates reported on Fig. 1b, are likely to be lower bounds of the corresponding natural probabilities. However, the probability of mixed forward and retro-signaling is likely to remain much less probable than the non-mixed signaling regimes because, as we shall see in the following, this kind of signaling properties reckon with parameter conditions that are somehow antagonist. Indeed, in the following sections we analyze in more details how some particular parameters influence the probabilities of these signaling types. Considerable attention is provided to the interpretation of some conditions on parameters which increase the probabilities of various signaling regimes, in terms of biochemical concepts like enzyme saturation and protein sequestration.

## Results

### A *n*-tier signaling cascade with an inhibitor

The system we deal with is a linear cascade made up of an arbitrary number *n* of cycles of single covalent modifications, e.g. single phosphorylation-dephosphorylation cycles. We also assume that the last level of the cascade may be altered by a kinase inhibitor, represented by a drug *D*, that blocks the action of the active protein by sequestering it into an inactive complex (Fig. 1a).

Our overall purpose is to investigate the working principles of such a generic cascade in terms of biochemical parameters like reaction rates and total enzyme concentrations. Thus, identify which parameter ranges are associated to specific signaling behaviors, which we call *regimes*. A signaling regime describes the way a cascade responds (significantly or negligibly) to the stimuli it is subjected to, namely the activator signal (at the top) and the inhibiting drug (at the bottom).

Practically, this means to measure two effects simultaneously: the impact of the activator on downstream proteins (dose-response curves) and the effect of the drug on the upstream proteins (drug-response curves). Specifically, we are interested in studying how the retroactive signaling propagates (from the (*n*−1)th to the first tier) and whether this is compatible with an efficient forward signaling relative to the *n*th tier.

In the following, we firstly show analytical results characterizing the most “natural" direction of propagation – the forward signaling – on a generic cascade of *n* tiers. We will show that this analytical approach provides some useful clues on elucidating key conditions that biochemical parameters should satisfy to observe an effective forward signaling of the cascade. On the other hand, the analytical approach soon becomes cumbersome, even by considering a homogeneous cascade (where parameters are the same for each tier). Therefore, in a second part of the Results we present a statistical investigation based on numerical computations, about all forms of signaling (forward and/or retroactive), for inhomogeneous cascades but with *n* fixed to 3.

### An iterative map for the cascade response functions

The system of equations describing the steady states of the *n*-cycle cascade depicted on Fig. 1a can be reformulated as a system of *n* iterative equations (details are shown in Methods) given by

1a $$\begin{array}{*{20}l} &s = \frac{x_{1}}{x_{1}+a_{1}} + \frac{b_{1} x_{1}}{(x_{1}+a_{1})\left(1-x_{1}- e_{2} \frac{x_{2}}{ x_{2}+a_{2}}\right) - c_{1} x_{1}}, \end{array} $$

1b $$\begin{array}{*{20}l} &x_{i-1} = \frac{b_{i} e_{i} x_{i}}{(x_{i}+a_{i})\left(1-x_{i}- e_{i+1} \frac{x_{i+1}}{x_{i+1}+a_{i+1}}\right) - c_{i} x_{i}},  \\ & 1<i< n,  \end{array} $$

1c $$\begin{array}{*{20}l} &x_{n-1} = \frac{b_{n} e_{n} x_{n}}{(x_{n}+a_{n})\left(1-x_{n}- d_{T} \frac{x_{n}}{x_{n}+a_{D}}\right) - c_{n} x_{n}},  \end{array} $$

with dimensionless variables, 1≤*i*≤*n*: $x_{i} = {Y_{i}^{1}}/Y_{iT},$ where ${Y_{i}^{1}}$ is the active protein, and *Y*_*iT*_ is the total protein concentration, so that *x*_*i*_ is the normalized active protein. Then the dimensionless parameters, 1≤*i*≤*n*, are defined as follows:

2 $$  \begin{array}{l} s = \frac{{k_{1}^{0}} \, Y_{0T}}{{k_{1}^{1}} E_{1T}}, \\ a_{i} = \frac{{K_{i}^{1}}}{Y_{iT}}, \quad b_{i} = \frac{{K_{i}^{0}}}{Y_{iT}}, \\ c_{i} = (1+ \frac{{k_{i}^{1}}}{{k_{i}^{0}}}) \frac{E_{iT}}{Y_{iT}}, \\ e_{i} = \frac{{k_{i}^{1}}\, E_{iT}}{{k_{i}^{0}}\,Y_{i-1,T}}, \\ d_{T} = \frac{D_{T}}{Y_{nT}}, \quad a_{D} = \frac{K_{D}}{Y_{nT}} \,, \end{array}  $$

where ${k_{i}^{0}}$ and ${k_{i}^{1}}$ are the catalytic rates of, respectively, the phosphorylation and dephosphorylation reactions; ${k_{i}^{0}}$ and ${k_{i}^{1}}$ are the Michaelis-Menten constants associated; *K*_*D*_ is the drug association-dissociation constant; *Y*_*iT*_, *E*_*iT*_ and *D*_*T*_ represent, respectively, the total concentrations of the proteins, the phosphatases and the drug.

This formulation is a generalization of the one presented for a 3-tier cascade in [18], extented to a cascade of arbitrary length *n*, with the addition of a drug.

In a more compact form, system (1) can be rewritten as:

3 $$\begin{array}{*{20}l} &s = \check f_{1}(x_{1},x_{2}),  \\ &x_{i-1} = f_{i}(x_{i},x_{i+1}), \quad 1<i< n, \\ &x_{n-1} = \hat f_{n}(x_{n},x_{n+1}=0,d_{T}) \,,  \end{array} $$

which represents the iterates of a second-order discrete map with boundary conditions given by signal *s* and *x*_*n*+1_.

We remark that, if the drug is absent, *i.e.d*_*T*_=0, then $\hat f_{n}(x_{n},x_{n+1}=0,d_{T})$ reduces to *f*_*n*_(*x*_*n*_,*x*_*n*+1_=0).

**Fig. 2:**
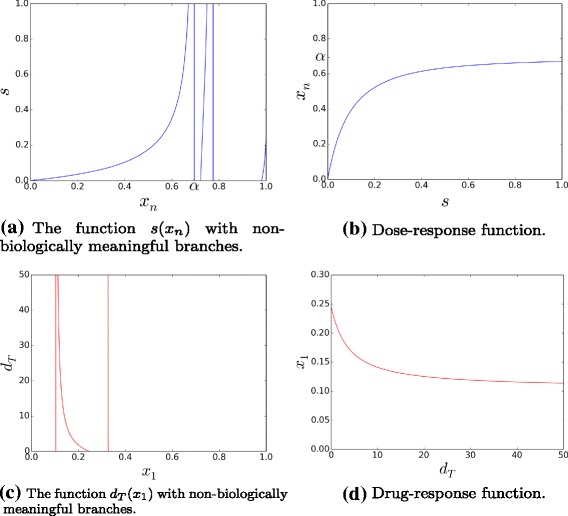
Examples of dose-response and drug-response functions computed from the iterative system (3) (see main text). Parameters for the dose-responses: *n*=3,*a*=1.6,*b*=0.8,*c*=0.05,*e*=0.7. Parameters for the drug-responses: *n*=3,*a*=2.2,*b*=0.0005,*c*=5.2,*e*=5.1

This iterative system allows us to obtain the dose-response function *x*_*n*_(*s*) as follows, provided that all the parameters (even *d*_*T*_) are fixed, except *s* : given *x*_*n*_∈[0,1), from Eq. 1c one can calculate *x*_*n*−1_, then from Eq. 1b one gets *x*_*n*−2_,…,*x*_1_, with *i* decreasing from *n*−1 to 2. Finally, *s* is computed by using 1a and one obtains function *s*(*x*_*n*_) which has typically several branches (see Fig. 2a), along the whole interval [0,1). Nevertheless, only one branch is biologically relevant [19], namely the one such that *s*(0)=0. This branch is defined on the domain [0,*α*), with *α* being the minimum value of *x*_*n*_ for which *s*(*x*_*n*_)→+*∞*. Thus, the biological dose-response curve is given by the restriction *s*(*x*_*n*_)|_[0,*α*)_ which is continuous and injective, so invertible on this domain. We denote such an inverse function as *x*_*n*_(*s*) (omitting the codomain restriction, for the sake of notation), see Fig. 2b.

Moreover, one can check that system (1) is consistent with the steady-state formulation derived in [19] (providing *d*_*T*_=0), where the authors particularly studied the properties of *s*(*x*_*n*_) as a rational function.

We also note that the maps $\check f_{1}, f_{i}$, and $\hat f_{n}$ are analytically invertible, leading to an inverse iterative system giving explicitly the inverse of the drug-response function *d*_*T*_(*x*_1_) (reported in Additional file 1), for a fixed signal *s*, *cf.* Fig. 2d.

### Dose-response functions: analytical characterization

The dose-response function *x*_*n*_(*s*) expresses how the activated protein in the last cycle of the cascade varies with the input signal *s* (proportional to the total enzyme *Y*_0*T*_ activating the first cycle of the cascade), given the quantity *d*_*T*_ fixed e.g. to zero.

For a cascade of *n*≥3 tiers, it is not possible to explicitly invert [the restriction of] function *s*(*x*_*n*_) to obtain an analytical expression of *x*_*n*_(*s*), because this requires finding the roots of a high-degree polynomial. Nonetheless, we illustrate how to provide qualitative knowledge of the non-saturating region, and quantitative estimation of the saturation value of the dose-response function.

In order to simplify the analytical expressions we assume that the parameters defined in Eq. 2 are the same for each *i*=1,2,…,*n*. We say that such a system is a homogeneous cascade, and in the rest of the paper we will omit to write the lower index *i*, if unnecessary. Some generalizations of our results to inhomogeneous cascades are given in Methods.

**Fig. 3:**
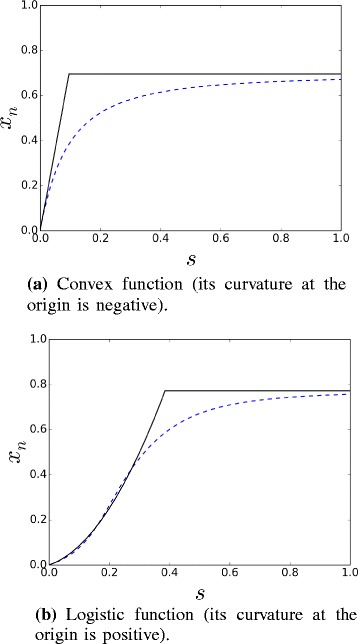
Sketch of typical dose-response functions. Dose-response curves *x*_*n*_(*s*) (dotted blue curves) and their piecewise approximations (solid black lines)

The strategy consists in approximating *x*_*n*_(*s*) piecewisely by matching analytical quantities (depending on parameters) evaluated at the origin (*s*=0) with the ones deduced at saturation (*s*→+*∞*), as illustrated in Fig. 3. Indeed the optimization of the efficiency of the forward signaling is based on the following approach.

The non-saturated part of the dose-response function is roughly described by a polynomial function (of first or second order according to the initial curvature sign – negative or positive, respectively), and the saturated part by a constant function.

More formally, we state that if the initial curvature *χ*=*xn*″(0)<0 (Fig. 3a), then:

4 $$  x_{n}(s) \sim \left\{ \begin{array}{ll} \sigma \, s \qquad &0 \leq s < p \\ \alpha &s \geq p \end{array},\quad \text{with } \,\, p = \frac{\alpha}{\sigma}\,, \right.  $$

where 0<*σ*<*∞* and 0<*α*<1 are defined as *σ*=*xn*′(0) and $\alpha = {\lim }_{s\to \infty } x_{n}(s)$.

In the other case, if *χ*>0 (Fig. 3b), then:

5 $$ \begin{aligned} x_{n}(s) & \sim \left\{ \begin{array}{ll} \sigma \, s +\frac{1}{2} \chi \, s^{2} \quad & 0 \leq s < q \\ \alpha & s \geq q \end{array} \right.,\quad \\ &\qquad\quad \text{with } q=\frac{-\sigma+\sqrt{\sigma^{2}+2\,\chi\,\alpha}}{\chi} \,. \end{aligned}  $$

Hence, a dose-response function can be sketched by a simple curve depending on the three parameters (*σ*,*χ*,*α*). Precisely, if *x*_*n*_(*s*) is convex (*χ*≤0), the dose-response only depends on its initial slope *σ* and on its asymptotic value *α*, while if *x*_*n*_(*s*) is logistic-like (*χ*>0), also the value of its initial curvature *χ* plays a role (if *χ* is large, the dose-response function will reach its asymptotic value for relatively low doses).

An advantage of our methodology is that, as shown below, *σ* and *χ* can be analytically calculated, and *α* estimated, in function of the biochemical parameters of the cascade. In turn, these results can be used to connect the parameters with standard characteristics of response functions, like the half maximal effective concentration *EC*_50_, or effective Hill coefficient *n*_*H*_. For example, simple estimates of the *EC*_50_ can be provided by the value of *s* such that *x*_*n*_(*s*)=*α*/2 in Eqs. 4 and 5, yielding the results:

6 $$  {EC}_{50} \sim \left\{ \begin{array}{ll} \frac{\alpha}{2\sigma} & \text{if}\ \ \chi < 0 \\ \frac{-\sigma+\sqrt{\sigma^{2}+\chi\,\alpha}}{\chi} & \text{if}\ \ \chi > 0 \end{array} \right.  $$

Effective Hill coefficients can be subjected to several definitions. A possible estimate may be obtained by computing twice the response coefficient $ s \, x_{n}^{\prime }(s)/x_{n}(s)$ at *s*=*EC*_50_.

Finding analytical conditions which maximize the response amplitude $\alpha = {\lim }_{s \to +\infty } x_{n}(s)$ is not trivial because, as mentioned before, the function *x*_*n*_(*s*) is generally speaking intractable. Nevertheless, for a homogeneous cascade, we prove that (see Methods) the asymptotic value of the dose-response curve *α*, is lower bounded by

7 $$  x^{*} = \frac{1-a-e-c + \sqrt{(1-a-e-c)^{2}+4(a-b\,e)}}{2} \,,  $$

with *a*,*b*,*c*,*e* defined in (2). *x*^∗^ is a fixed point of the map *f* defined at Eq. 3 as *f*_*i*_. Thus, since *x*^∗^≤*α*, requiring a large *x*^∗^ is sufficient to have a large amplitude *α* and therefore this is a sufficient condition to fulfill the criteria to promote an efficient forward signaling.

Moreover, for homogeneous parameters, the initial slope of the dose-response is

8 $$  \sigma = \frac{a}{1+b} \left(\frac{a}{b \,e} \right)^{n-1} \,,  $$

and the initial curvature for *n*=3, and *d*_*T*_,*a*_*D*_ fixed, is

9 $$ \begin{aligned} \chi &= - \bigg(\frac{b^{2}\,e^{2}}{a^{2}} \big(a+b\,(a+c-1)-1 \big) + \frac{a(1+b) d_{T}}{a_{D}} \\ & \qquad + \frac{(1+b)e}{a} \big(a+b\,(a+c-1) \big) \\ & \qquad + (1+b) (a+c-1) \bigg) \frac{a}{1+b} \left(\frac{a}{b\,e} \right)^{4} \,. \end{aligned}  $$

In Methods we derive these and more general formulas for arbitrary *n* and inhomogeneous parameters.

Therefore we have shown how the parameters (*σ*,*χ*,*x*^∗^) characterizing the sketchy dose-response curves (Fig. 3) can be expressed as functions of the cascade parameters (*a*,*b*,*c*,*e*).

Now we want to state analytical conditions on parameter sets, that enhance forward signaling. As simple criteria, we say that a parameter set provides efficient signaling if it maximizes *σ* and *α* if *χ*<0, or maximize *χ* and *α* if *χ*>0. Table 1 sorts the sufficient conditions deduced from these criteria, thus optimizing the downstream propagation in a homogeneous cascade of length 3.

**Table 1:** Sufficient conditions to optimize the forward signaling. The second column reports combined parameter ranges able to enhance the forward response for convex (*χ*≤0) and logistic-like (*χ*>0) curves, deduced analytically from the criteria of efficient forward signaling. The third column refers to conditions obtained below, with a numerical method based on a random parameter sampling and maximizing the likelihood of these parameters with respect to the forward signaling (See Discussion section)

Parameters	Suff. conditions for		Maximizing likelihood
	*χ*≤0	*χ*>0	(cf. discussion below)
$a = \frac {K^{1}}{Y_{T}}$	>1	≪1	*a*_3_≫1
$b = \frac {K^{0}}{Y_{T}}$	<1	<1	*b*_*i*_≪1
$1/e = \frac {k^{0}\,Y_{T}}{k^{1}\,E_{T}}$	≫1	≫1	1/*e*_*i*_>1
$c-e = \frac {E_{T}}{Y_{T}}$	≪1	≪1	*c*_*i*_−*e*_*i*_<1
$\frac {a}{b} = \frac {K^{1}}{K^{0}}$	>1	≪1	*a*_*i*_/*b*_*i*_>1

In order to interpret the results summarized in this table, let us remember that for an enzymatic reaction, the enzyme is said to be *saturated* by its substrate when the Michaelis-Menten constant is small compared with the total concentration substrate. On the other hand, if the total enzyme concentration is not small compared with the total substrate, the free substrate is expected to be *sequestrated* by the substrate-enzyme complex. Moreover, in the case of an enzymatic cycle, we call *activation* parameter, the ratio between the maximal reaction rates of the two enzymes phosphorylating and dephosphorylating a protein.

In light of this terminology, the first line of Table 1 shows that convex and logistic-like dose-responses are characterized respectively by non-saturation and high saturation of the phosphatases. A similar observation but concerning a single enzymatic cycle was reported in [20]. So this finding turns out to be generalizable to a whole cascade. The second line of the Table indicates that a moderate saturation of the kinase is also a condition that promotes forward signaling, whatever the curvature of the dose-response is. Finally, the third and fourth lines of the same Table reveal two general features enhancing forward signaling: high activation of the enzymatic cycles as well as non-sequestration of the active proteins by the phosphatase. This latter result is in agreement with the ones discussed in [3], where the authors compare the effect of sequestration and non-sequestration on logistic-like dose-responses in a MAPK (Mitogen Activated Protein Kinase) cascade.

### Drug-response functions

By *retroactivity* we mean that a perturbation, applied at a certain level of a cascade, propagates upstream, thus altering the previous tiers. In our system, this perturbation is initiated by a compound *D* (called *drug*) inhibiting the activated protein at the *n*th tier. Our goal is to study the effect of such a perturbation on the upstream levels as a function of some normalized drug concentration *d*_*T*_, assuming that the signal *s* at the top of the cascade is constant and fixed at a high value.

We classify the retroactivity according to its maximal propagation range, so that we call *retroactivity of order k* (1≤*k*≤*n*−1), the variation of the activated protein at the (*n*−*k*)th level as a function of the drug concentration, described by the function *x*_*n*−*k*_(*d*_*T*_), which we refer to as *drug-response function*. In particular the highest order of retroactivity in a linear cascade corresponds to the response curve *x*_1_(*d*_*T*_).

As shown by [19], a perturbation propagates upstream in an alternated way so that, at level *n*, the amount of activated protein decreases, at level *n*−1 it increases, then it decreases at level *n*−2 and so on, up to the first level. It follows that function *x*_1_(*d*_*T*_) is increasing if *n* is even and decreasing if *n* is odd. Moreover, retroactivity is overall attenuated in long cascades, but can propagate and amplify its effect for *n* sufficiently small, e.g. equal to three [11].

Here, although we derive the drug-response functions in an iterative formulation inverting the map in (3) (*cf.* Additional file 1), the study of the derivatives at the origin *d*_*T*_=0 becomes too complicated to be performed analytically, as we did for dose-response functions *x*_*i*_(*s*) for 1≤*i*≤*n*. The main drawback is that, for *d*_*T*_=0, we have *x*_*i*_(*d*_*T*_)≠0 for any 1≤*i*≤*n*, so that the expressions of the initial slope and curvature of function *x*_*i*_(*d*_*T*_) do not simplify, as it does in the case of the dose-response functions.

Therefore, in the following, we compute the amplitude of the drug-response function by means of a numerical approach, for *n*=3 fixed, considering *Δx*_*i*_ the difference between the values *x*_*i*_(*d*_*T*_=0) and ${\lim }_{d_{T} \to +\infty } x_{i}(d_{T})$, for *i*=1,2. In these computations *x*_*i*_(*d*_*T*_=0) corresponds to the limit of the dose-response function *x*_*i*_(*s*), for *s*→+*∞*, while ${\lim }_{d_{T} \to +\infty } x_{i}(d_{T})$ corresponds to the limit of the dose-response function *x*_*i*_(*s*), for *s*→+*∞*, for the same cascade but composed only by the first *n*−1 levels (as a result of the total sequestration of the last-level active protein by the drug, at steady state).

### Random sampling of the parameter space

In this section we consider a 3-tier signaling cascade and numerically estimate the probability of finding one of the possible signaling regimes, in function of key system’s parameters. We define 8 signaling regimes and denote them by (*jkl*), with *j*,*k*,*l*∈{0,1}, where:

- *j*=1 if the amplitude of the drug-response curve *x*_1_(*d*_*T*_) is larger than 5 %, *j*=0 otherwise;
- *k*=1 if the amplitude of the drug-response curve *x*_2_(*d*_*T*_) is larger than 5 %, *k*=0 otherwise;
- *l*=1 if the amplitude of the dose-response curve *x*_3_(*s*) is larger than 50 %, its slope at the origin is larger than 1 or the curvature should be at least 1, and *l*=0 otherwise.

For instance, the signaling regime (001) corresponds to parameters associated to a cascade which exclusively exhibits forward propagation, while (110) is for exclusive retroactive propagation. Signaling regimes of type (*j*1*l*) will be said to possess *first order* retroactivity, whereas regimes of the type (1*kl*) will be said to have *second order retroactivity*. Later, to discuss the notion of signaling motifs (*cf.* Fig. 5), it will be convenient to consider *hybrid* signaling regimes like (1*k*0), where *k* is not determined (*k*=0,1). Finally, (000) is the *anti-signaling* regime, as it denotes the absence of any type of signaling response.

By performing a random sampling of the biochemical parameters, like reaction rates and total concentrations (see Methods), we have assessed the probability of each regime, Fig. 1b.

### Likelihoods of parameters for the signaling regimes

Our numerical investigation considers dimensional and dimensionless parameters. The dimensional parameters are coefficients of the steady state Eqs. 2, such that total kinase concentrations, total phosphatase concentration, Michaelis-Menten constants, etc... In the sequel the *dimensional* parameters are simply called biochemical parameters. On the other hand, the *dimensionless* parameters, say *λ*, are ratios of these biochemical parameters, such as fractions of total phosphatase over total kinase concentrations, and so on.

We perform a random sampling of the biochemical parameters, with 18 dimensionless parameters being computed from these first ones. Afterward, we estimate the probability distributions (histograms) of all the dimensionless parameters *λ* for each signaling regime (*jkl*) defined above. Then, using Bayes’ formula (see Methods, Eq. 20) we deduce the probability of finding a given signaling regime (*jkl*), provided that a given parameter value *λ* is picked up. This quantity, seen as a function of *λ*, is called *likelihood*. We denote the likelihood normalized by its maximum value by *L*_*jkl*_(*λ*) (*cf.* Eq. 21). In order to identify the region of the parameter space enhancing a given type of signaling regime, our main strategy is to look for the values of dimensionless parameters that maximize the likelihood for the considered signaling regime. The result can generally be expressed as inequality conditions that should be satisfied between biochemical parameters.

**Fig. 4:**
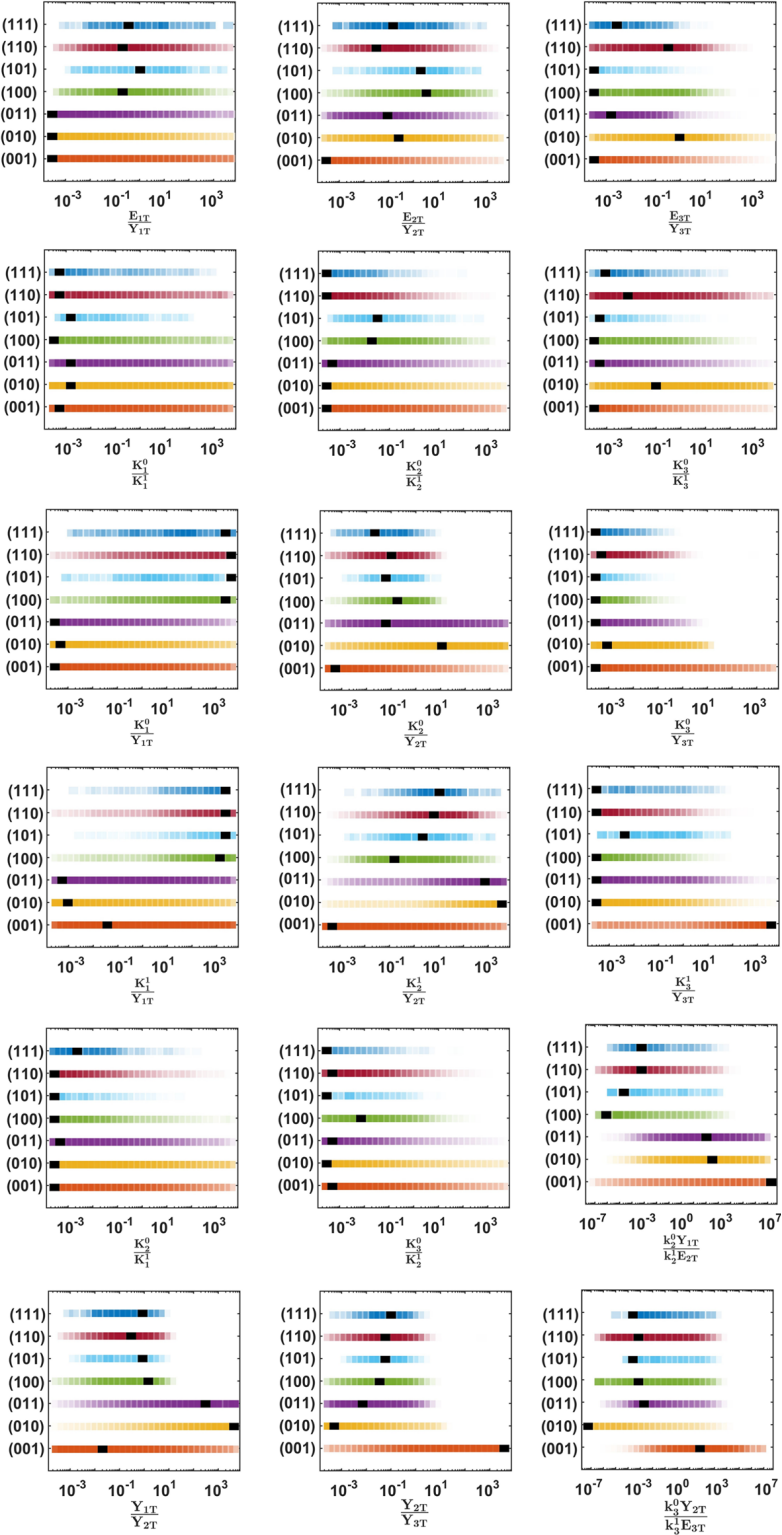
Normalized likelihoods of 18 dimensionless parameters (proportional to color intensities), superimposed for all signaling regimes (*j* *k* *l*)≠(000)

On Fig. 4 each colored band shows the normalized likelihood of one given parameter, obtained for every signaling regimes (*jkl*). Thus, the intensity of the color in each band is proportional to the probability that a specific regime (*jkl*), with *j*,*k*,*l*∈{0,1}, occurs, in function of a given dimensionless parameter, all the biochemical parameters being chosen at random in a log-uniform distribution. In Additional file 2, the numerically computed likelihoods are also represented as curves with estimated errorbars.

We report here on our analysis of the likelihood variations relative to a cascade with inhomogeneous parameters. We mainly proceed by visual inspection of the likelihood variations. In this way we can classify the 18 dimensionless parameters into two classes. The first class corresponds to 9 parameters for which similar ranges optimize the probability of any type of signaling (*jkl*), (000) excluded. In the second class we put the 9 parameters left, which exhibit likelihood variations being useful to discriminate between the signaling regimes, and specifically related to the probability of a given regime.

After a visual analysis of all the parameter likelihoods we conclude that the following 9 dimensionless parameters form a first class : *E*_*iT*_/*Y*_*iT*_, ${K_{i}^{0}} / {K_{i}^{1}}(i=1,2,3)$, $K_{i+1}^{0} / {K_{i}^{1}}(i=1,2)$, ${K_{3}^{0}} / Y_{3T}$. For instance, we see that the range enhancing the likelihood for any regime (*jkl*)≠(000) corresponds to choose ${K_{3}^{0}} / Y_{3T}$ as small as possible. On the contrary choosing ${K_{3}^{0}} / Y_{3T}$ large hinders any type of signaling regime.

On the other hand, as discussed below, the following 9 dimensionless parameters enable to discriminate amongst the various types of signaling forms: $({k_{i}^{0}} Y_{i-1,T})/({k_{i}^{1}} E_{iT})$ (*i*=2,3), *Y*_*iT*_/*Y*_*i*+1,*T*_, $Y_{iT} / {K_{i}^{1}}$, $Y_{iT} / {K_{i}^{0}}$ (*i*=1,2), $E_{iT}/{K_{i}^{1}}$ and $E_{iT}/{K_{i}^{0}}$ (*i*=2,3).

In the following two subsections we first report on conditions about the biochemical parameters that promote indistinguishably any form of signaling regimes. Then we discuss the role of other conditions on biochemical parameters specific to each regime.

#### General parameter conditions promoting signaling

Some constraints on parameters appear to be common to any signaling regimes. Moreover, when these conditions are chosen opposite, the probability of getting any type of signaling tends to be negligible. These conditions are listed as follows :

(i) *E*_*iT*_≤*Y*_*iT*_ (*i*=1,2,3) : at each tier of the cascade, the sequestration of the proteins by their phosphatase is absent or moderate.
(ii) ${K_{i}^{0}} \ll {K_{i}^{1}} \quad (i=1,2,3)$ : enzymatic asymmetry in cycle *i* : the affinity of the kinase for its substrate should be larger than the one of its phosphatase.
(iii) $K_{i+1}^{0} \ll {K_{i}^{1}} \quad (i=1,2)$ : enzymatic asymmetry at the junction of tiers *i* and *i*+1 : the affinity of an activated protein for its substrate, *i.e.* the next protein in the cascade, is larger than for its own phosphatase.
(iv) ${K_{3}^{0}} \ll Y_{3T}$ : the second kinase ${Y_{2}^{1}}$ is saturated.

#### Regime-specific parameter conditions

The various signaling regimes are actually determined by a specific combination of parameters concerning cycle activation, protein sequestration and enzyme saturation. Here we discuss the effect of the second class of parameters that specifically favor some types of regime.

(v) $1/e_{i} = ({k_{i}^{0}} Y_{i-1,T})/({k_{i}^{1}} E_{iT}) \quad (i=2,3)$ : cycle deactivation (*e*_*i*_>1) gives rise to retroactive signaling. Notably, retroactivity appears whenever the third tier is deactivated (*e*_3_>1). In this case, its effect can be either local (limited to the second tier, *cf.* regimes (01*l*)) if the second tier is activated (*e*_2_<1 so preventing an upper propagation), or global (*e*_2_>1, affecting both the previous tiers, *cf.* regimes (1*kl*)) if also the second tier is deactivated.
(vi) *Y*_*iT*_/*Y*_*i*+1,*T*_ (*i*=1,2) : 4 distinct protein progressions typify the retroactive regimes, *i.e.* 00*l* (no retroactivity), 01*l* (first order retroactivity), 10*l* (second order retroactivity), and 11*l* (first and second order retroactivity), with *l*∈{0,1}. This parameter is associated to sequestration. More particularly, the sequestration of the third inactive protein by its kinase (*Y*_2*T*_/*Y*_3*T*_≫1) prevents any retroactive propagation (regimes (000) and (001)). The sequestration of the second inactive protein by its kinase (*Y*_1*T*_/*Y*_2*T*_≫1) promotes first order retroactivity, *i.e.* regime (01*l*). Inversely, non-sequestration (*Y*_1*T*_/*Y*_2*T*_∼1) induces second order retroactivity, *i.e.* regime (1*kl*).
(vii) ${K_{i}^{1}}/Y_{iT} \quad (i=1,2,3)$ : phosphatase saturation (${K_{i}^{1}}/Y_{iT}<1$) or non-saturation (${{K_{i}^{1}}/Y_{iT}\geq 1}$) marks out the appearance of second order retroactivity and forward signaling. In particular, saturation (respectively non-saturation) of the first phosphatase is associated to negligible (respectively significative) second order retroactivity, *i.e.j*=0 (respectively *j*=1). Instead, saturation (respectively non-saturation) of the third phosphatase is associated to significative (respectively negligible) forward propagation, *i.e.l*=1 (respectively *l*=0). Moreover, saturated phosphatase at the second tier marks the complete absence of retroactivity (regimes (000) and (001)).
(viii) ${K_{i}^{0}}/Y_{iT} \quad (i=1,2)$ : saturation (respectively non-saturation) of the input signal, ${K_{1}^{0}}/Y_{1T}<1$ (respectively ${K_{1}^{0}}/Y_{1T}\geq 1$), characterizes a negligible (significative) second order retroactivity, *i.e.j*=0 (respectively *j*=1). Non-saturation of the kinase activating the second tier (${K_{1}^{0}}/Y_{1T}\geq 1$) is typical of first order retroactivity (regimes (010) and (011)).

This analysis leads to the following criteria to enhance to probability *P* of significant response at each stage *j*,*k*,*l* within a signaling regime (*jkl*):

(I) *P*(*j*=1) is enhanced if and only if $Y_{1T} \ll {K_{1}^{1}}$.
(II) *P*(*k*=1) is enhanced if *e*_2_<1 and *e*_3_>1.
(III) *P*(*l*=1) is enhanced if and only if *E*_3*T*_≪*Y*_3*T*_.

Moreover we claim the following *necessary conditions*:

(IV) If (I) holds then *e*_2_>1 (notably, *e*_2_>1 for (110) and (111), and *e*_2_≫1 for (100) and (101)).
(V) If conditions of (II) holds then *j*=0.

### Graphical representation of the signaling regimes

In the previous section, conditions on biochemical parameters which characterize the signaling regimes, have been deduced from the likelihoods of dimensionless parameters. At this stage it is difficult to imagine how, among the various signaling regimes, such conditions are similar or different from each other. Therefore in this section we introduce a method to graphically depict these conditions and visually link them to the cascade signaling properties. The idea is to associate a pictorial code to the parameters in such a way that their graphical representation conveys qualitatively the corresponding signaling regimes.

**Fig. 5:**
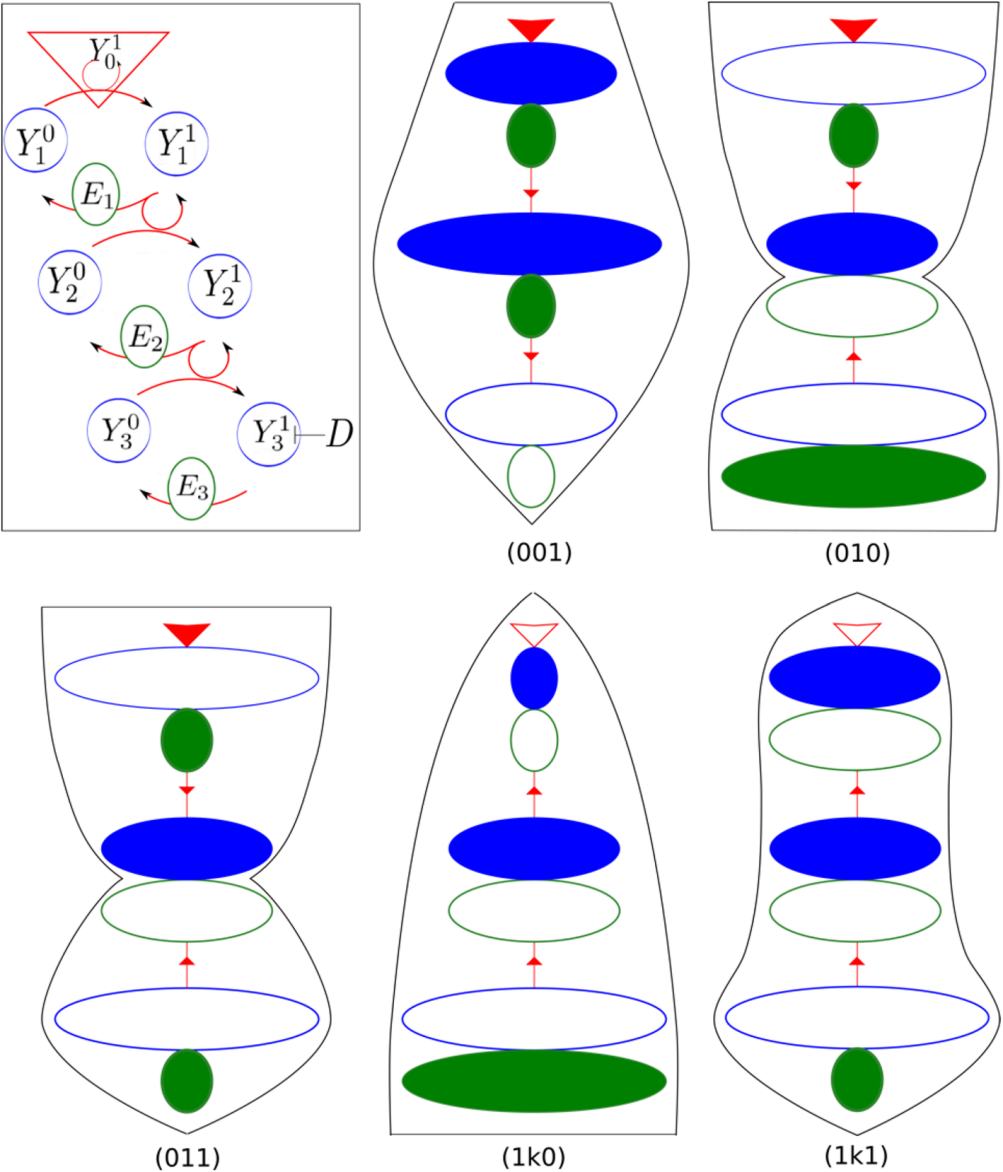
Motifs representing qualitatively conditions on the cascade’s main parameters (top left) optimizing the likelihood of each signaling regime. Graphical codes for the biochemical species: the triangle corresponds to input signal ${Y_{0}^{1}}$, the ellipses to total proteins *Y*_*iT*_ or total phosphatases *E*_*iT*_, and the number and direction of the arrows on the segments represent the intensity of cycle activation (if downward arrows) or cycle deactivation (if upward arrows). Color: blue is for kinase, green for phosphatase; size: total concentration of the species; texture: shaded means saturated, empty unsaturated. Drawing of motifs is detailed in Additional file 3

The method is based on the visual examination of the maximal likelihoods of dimensionless parameters (Fig. 4). Its application, detailed in Additional file 3, leads to draw the 5 signaling motifs depicted on Fig. 5, which are associated respectively to signaling regimes (001), (010), (011), (1*k*0),(1*k*1). (Recall that the two latter denote hybrid signaling regimes, with non-determined *k*=0 or 1). The procedure, providing conditions on biochemical parameters which optimize the probability to observe such regimes, consists in 5 steps described below.

1. Fix the size of *Y*_2*T*_ and *Y*_3*T*_, then *Y*_1*T*_ (blue ellipses) as follows.
  - If *Y*_2*T*_≫*Y*_3*T*_ then draw *Y*_2*T*_ large and *Y*_3*T*_ medium. Then, if *Y*_1*T*_≪*Y*_2*T*_, draw *Y*_1*T*_ medium.
  - If *Y*_2*T*_<*Y*_3*T*_ (with a magnitude difference of 100 at most) then draw *Y*_2*T*_ medium and *Y*_3*T*_ large. Then if *Y*_1*T*_∼*Y*_2*T*_, draw *Y*_1*T*_ medium and if *Y*_1*T*_<*Y*_2*T*_, draw *Y*_1*T*_ small.
  - If *Y*_2*T*_≪*Y*_3*T*_ (with a magnitude difference larger than 100), then draw *Y*_2*T*_ medium and *Y*_3*T*_ large. Then if *Y*_1*T*_≫*Y*_2*T*_, draw *Y*_1*T*_ large.
2. Fix the size of the *E*_*iT*_’s (*i*=1,2,3).
  - If *E*_*iT*_≪*Y*_*iT*_ then draw *E*_*iT*_ small.
  - If *E*_*iT*_∼*Y*_*iT*_ then draw *E*_*iT*_ of the same size of *Y*_*iT*_.
  - If *E*_*iT*_≫*Y*_*iT*_ then draw *E*_*iT*_ extra large.
3. Represent signaling connectivity (*i*=1,2).
  - If *e*_*i*_>1, draw one arrow pointing upward.
  - If *e*_*i*_<1, draw one arrow pointing downward.
4. Empty/full shapes.
  - If $Y_{iT} \gg {K^{0}_{i}}$ for *i*=1, fill in the red triangle.
  - If $Y_{iT} \gg {K^{0}_{i}}$ for *i*=2,3, fill in the (*i*−1)th blue ellipse.
  - If $Y_{iT} \gg {K^{1}_{i}}$ for *i*=1,2,3, fill in the *i*th green ellipse.
5. Draw the motif contour following the largest ellipses. Instead of a straight line, the contour will start with a cusp if the red triangle is empty while the first blue solid ellipse is full, and it will end with a cusp if step 2.a is fulfilled.

Using the last step of the procedure, the contour line traced around each motif follows the total proteins’ progression and start or ends with a cusp according to the presence of, respectively, second-order retroactivity or forward signaling. Moreover the flow of signal propagation is directed by arrows pointing upward or downward, according to the *e*_*i*_’s (*i*=2,3). As a matter of fact it appears that the final picture can be easily interpreted in terms of signaling regimes (*jkl*), with the convention that a cusp or a bottleneck in the figure contour means a successful amplitude response at the corresponding tier. Therefore one main benefit of the procedure is to automatically turn the parameter conditions analyzed in last section, into qualitative motifs which are easy to read out in terms of their signaling properties. Conversely, these patterns are easier to remember than a list of conditions, and can thus be used as a tool to recover the criteria for each of signaling schemes.

In conclusion, these pictures show that retroactivity is promoted by four features: the last tier is deactivated (*e*_*i*_>1), the second active protein is sequestrated, the second phosphatase is non-saturated and the third inactive protein is not sequestrated.

In particular, second-order retroactivity is enhanced if the first protein is saturated, and the input signal and the first phosphatase are not; inversely for first-order retroactivity.

A forward response (*l*=1) is (most likely) negligible if the last active protein is not sequestrated by its phosphatase (*E*_3*T*_≪*Y*_3*T*_). On the other hand, regime (001) is favored by a saturated phosphatase at the second tier.

## Discussion

Kinase cascades are a key component of the living cell systems biology. Relying on several biochemical parameters, the standard functioning of a cascade is to transmit forward signals between the top and the bottom of the pathway. Nevertheless, the possibility of transmitting information backward in the cascade, due to sequestration effects of the enzymes, has been demonstrated by several studies (see Background). However, the backward – or what we called *retroactive* – signaling is not considered in the current literature as a standard property that cascades should possess or avoid. Therefore our main question, that was raised at the start of this paper, was to study how cascade parameters match when promoting one or the other mode of signaling. How similar or different were the parameter sets that enabled forward or retroactive signaling? Were they incompatible? Could we characterize these parameter sets in terms of biochemical concepts such as enzymatic cycle activation, enzyme saturation or sequestration?

These questions were theoretically approached with analytical and numerical methods. Both ways showed advantages and limitations. One advantage of the analytical trail was to enable a study of the dose-response function for cascades with arbitrary length *n*. With this general approach, however, we usually had to limit our analysis to homogeneous cascades (*i.e.* same parameters at each tier), although some results can be generalized to inhomogeneous parameters. Nevertheless we showed that the dose-response function could be represented as the iteration of an explicit rational function. This iterative structure allowed us to compute analytical properties of the response function, like for example its first and second derivatives at the origin. These computations, together with the determination of a lower bound of the maximal value of the response function, revealed to be invaluable in discussing conditions on the biochemical parameters that optimize forward signaling in homogeneous cascades. These results, summarized on Table 1, were corroborated later by the numerical computations, that explored also cascades with inhomogeneous parameters.

However, contrary to the dose-response curve, the analytical study of the drug-response function was out of reach with our analytical tools. Thus the advantage of the considered numerical approach was to extend our analytical investigation to inhomogeneous cascades, and to study the drug-responses and retroactivity properties. As the number of parameters increases with the length of the cascade, one limitation of this approach, however, was to focus only on the case of a 3-tier cascade. The methodology was based on a random scan of the parameter space of such cascades, and a subsequent classification of parameter ranges according to their ability to produce dose-response or drug-response curves satisfying minimal criteria (e.g. regarding the response amplitudes). A bayesian analysis of a set of dimensionless parameters (ratios of two biochemical parameters) allowed us to infer biochemical conditions favoring a specific signaling regime.

In what concerns the forward signaling regime, although both analytical and numerical approaches are very different, they point towards the same conditions, as reported in Table 1. In this table the second column reports analytical conditions enhancing forward signaling in a homogeneous cascade, whereas the third column describes conditions maximizing the likelihood of inhomogeneous parameters relative to the forward signaling regime. The obtained conditions are found to be compatible in all cases, sometimes with some dependence of the cascade layer. In particular: (i) the affinity of the substrate for the kinase should be larger than its affinity for the phosphatase, creating what we call *enzymatic asymmetry*, (ii) the phosphatases should be in small amount compared with their total substrate, and (iii) the maximal rate of phosphorylation should be larger than the maximal rate of the dephosphorylation.

In the context of theoretical studies of signaling systems, the use of random scans of biochemical parameters, in order to determine parameter ranges or conditions giving a sought property, has been considered by several authors. Typically these authors uniformly scanned dimensional parameters [21–23], or dimensionless parameters [12, 24, 25]. Often, the use of dimensionless parameters is motivated by a procedure of non-dimensionalization of the kinetic or of the steady state equations. In our study, we chose a random sampling of dimensional parameters. We believe that in general it leads to a better interpretration of the results. The reason is that if we start by scanning the dimensionless parameter space then, because of the change of variables between the dimensionless and dimensional parameters, then a uniform probability distribution of the dimensionless parameters is transformed into a *non-uniform* density for the dimensional parameters. Then the conclusions drawn from the statistical results must be associated with some non-uniform distribution of dimensional parameters. These non-uniform distributions might be non-trivial to figure out, and overall rather arbitrary, as there exist several ways to design non-dimensionalizing procedures. Therefore, although in our methodology we statistically analyze a set of dimensionless parameters in view of biochemical interpretation, dimensional biochemical parameters were first randomly sampled (in log-uniform distribution), the dimensionless parameter being deduced afterwards from these samplings.

In our study, amongst the 18 sampled biochemical parameters, 12 dimensions corresponded to chemical concentrations and 6 dimensions to reaction rates (with dimension of inverse of time). They were all chosen in the interval [10^−2^,10^2^], thus considering 4 orders of magnitude [−2,2] in uniform log10 scale. The interpretation of the results depends yet on the choice of the reference unit concentration (the “0" in log scale). For example, if the reference dimensional concentration is chosen as 0.1 *μ*M, this leads to interpreting the scanned intervals as the range [1 nM, 10 *μ*M], which seems reasonable as intracellular concentrations [15]. However this is just an example and the choice of the reference unit concentration remains a degree of freedom in our numerical methodology.

On Fig. 1b we reported estimated probabilities of four signaling modes of the cascade, *i.e.* no-signaling, forward signaling, retrosignaling, or simultaneous forward and retro-signaling. Obviously, the absolute value of these numbers depend on the arbitrary thresholds on response amplitudes, that we fixed to assess the occurence of these signaling modes. To give evidence of the effect of changing these thresholds on the probabilities of signaling, Table 2 reports the occurence frequency of the 8 considered signaling regimes, for two different choices of thresholds (distinguishing further amongst the four main signaling modes of the cascades, the cases of retroactivity of first and of second order). Although the actual numbers are different when the thresholds are increased, we observe constancies.

**Table 2:** Probabilities of the signaling regimes according to criteria stated in Results, with 2 different choices of thresholds for the response amplitudes. Threshold 1: *Δ* *x*_1_,*Δ* *x*_2_>5 *%*, *Δ* *x*_3_>50 *%*. Threshold 2: *Δ* *x*_1_,*Δ* *x*_2_>10 *%*, *Δ* *x*_3_>75 *%*

Regimes	Probabilities (in %)	
	Threshold 1	Threshold 2
(000)	66,11 ± 0.05	72,15 ± 0,04
(001)	19,74 ± 0.04	16,78 ± 0,04
(010)	11,35 ± 0.03	9,73 ± 0,03
(011)	1,89 ± 0.01	0,828 ± 0,009
(110)	0,474 ± 0.007	0,228 ± 0,004
(100)	0,383 ± 0.006	0,271 ± 0,005
(111)	0,035 ± 0.002	0,0068 ± 0,0008
(101)	0,027 ± 0.002	0,0087 ± 0,0009

Obviously, the most probable parameters are always the “non-signaling" cases (000), and the most probable non-trivial signaling regime is the pure forward signaling (001). Then the probability of signaling regimes always decreases markedly between the cases of retrosignaling of first and second order. In particular one observes that the probabilities of regimes (1*k*1), admitting both forward and retro-signaling of order 2, are the smallest ones, and get smaller and smaller with higher thresholds. Therefore as a whole, these numbers confirm the general tendency of our numerical results: system’s parameters enabling bidirectional signaling correspond to the most unlikely cases.

This property can also be characterized in a quantitative way by computing the conditional probability that bidirectional signaling occurs, knowing that the system exhibits at least one regime of signaling (so excluding (000)). This conditional probability is found to be 6 *%* with the thresholds corresponding to column 1 of Table 2, and drops to 3 *%* with data in the second column. Moreover we checked that it decreases from 6 to 5 *%* when the uniform ranges of biochemical parameters are extended from [−2,2] to [−2.5,2.5] (data not shown). Therefore we can conclude that requiring both response amplitudes of direct and retrosignaling to be large leads to an antagonism in the parameter sets achieving both requirements. The overall combination of forward and retro-signaling appears to be still more rare. This conclusion answers one of the principal question addressed by this study.

Another question addressed in Background was to characterize, for each signaling regimes, conditions on biochemical parameters that promote the corresponding regimes. We have achieved such characterization by looking, for each signaling regime, at the likelihood of dimensionless parameters associated with the concepts of enzyme saturation, enzyme sequestration, kinase/phosphatase affinity asymmetry, or enzymatic cycle activation. Here also the choice of thresholds on the amplitude responses has a mild importance on the conclusions we point out, since we highlighted parameter conditions that maximized the (normalized) likelihood of parameters, for each signaling regime. By considering higher thresholds, we have checked that we keep same ranges where the likelihood is maximized, even if the actual value of the likelihood is reduced (see Additional file 4).

Therefore the obtained conditions on parameters, as reported in Results, could be useful to distinguish parameter sets optimizing the probability to get the various regimes of signaling. These conditions are graphically represented in Fig. 5, with a motif coding that is suggestive of the corresponding form of signaling. The motif can be drawn following an algorithm, which could be implemented in an automatic way, in order to facilitate the association between a set of parameters and the probable signaling regimes that could be observed with them. On the other hand, level-specific parameter conditions can be related to the probability for a distinct stage to respond or not to upstream or downstream perturbations, as claimed in our criteria (I)–(III).

**Fig. 6:**
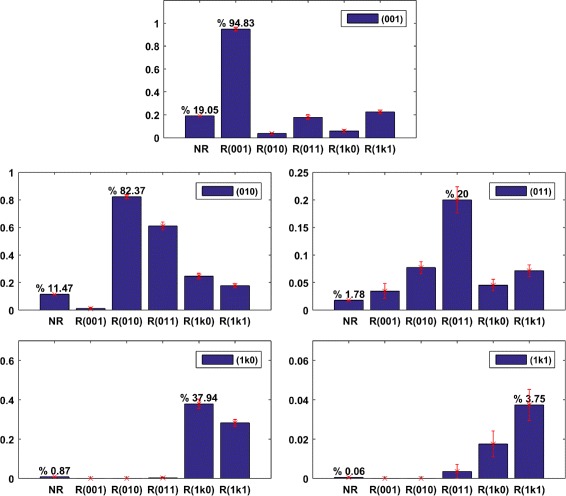
We consider the 5 signaling regimes corresponding to motifs of Fig. 5, as well as associated parameter restrictions characterizing each of them (see details in Discussion). Then, each panel displays the probabilities of a given signaling regime in function of the considered parameter restrictions. Consistently, the probability to get a given signaling regime is maximized by choosing the parameter restrictions characterizing it, and this maximum is significantly higher than the probability obtained from a log-uniform distribution of biochemical parameters without any restriction (cf. first bar of the histogram, NR)

These graphs embody also a method to restrict parameter space in order to enhance the probability of the main modes of signaling, forward, or retroactive, or both. In order to control how the probability of each signaling regime is optimized by choosing parameter values around the likelihood maxima, we divided the ranges of all the 18 parameters in three intervals: high values, medium values, low values. Each parameter was restricted to one of the three intervals depending on where its likelihood was maximized. As there is one restriction for each parameter, the intersection of those restricted intervals could leave, at the end, no more than 0.1 % of the initial number of simulation sets. The new probabilities of each regime were computed and compared with the former simulation sets in order to see how likely is a given regime if we restrict the ranges of the biochemical parameters. The results are summarized on Fig. 6 (see also Additional file 5). In a consistent way, these histograms show that the probability to observe a given signaling regime, among one of the 5 motifs of Fig. 5, is maximized by choosing parameter restrictions associated with the corresponding signaling regime. In particular we see that the optimized probabilities are much higher than in the unrestricted case (top panel). Only the probability of regimes involving both forward signaling and retroactive signaling of order 2 remains relatively small (3,7 %), which evokes once again the scarcity of bidirectional signaling.

## Conclusion

Living organisms rely strongly on biochemistry. Indeed signaling pathways are meant to transduce physical or chemical stimuli of the external world, relevant to living cells, into variations of activated biochemical species. In this paper, in addition to the common *dose-response* of a signaling cascade, we have also considered the bottom-up *drug-response* that discloses, when it exists, a retroactive capability of the signaling cascade. Thus signaling cascades can be categorized into 4 modes of responses, according to the existence or not of forward or of retroactive responses. An example of this classification with estimated probabilities was given in Background in Fig. 1b. This result was further discussed in the previous section. Although the quantitative aspects of our classification depends on some arbitrariness (e.g. thresholds of the amplitude responses for categorization, or the method of random scan, discussed below in Methods), our work confirmed the initial intuition that was exposed in Background: there is an opposition between the parameter sets of the cascade that promote forward (*i.e.* usual) signaling, and parameters that enable retroactive regimes, *i.e.* backward signaling.

Thus our main conclusion is that the parameter sets allowing both modes of responses, forward and retroactive, occur rarely. Actually, signaling cascades are generally studied uniquely for their forward signaling ability. For instance in cancer etiology, attention is focused on over-activation of kinases in signaling pathways involved in cell proliferation, such as Mitogen Activated Protein Kinase cascades (MAPK). When these cascade pathways are deregulated in this manner, this means that their forward signaling properties are very effective. Moreover in this case, cancer therapies are based on kinase inhibition, which is described by the drug binding term in our mathematical modeling. Therefore, our main result conforts the point of view that in using these therapies, retroactive properties of signaling cascade can be neglected most of the time, eventhough rare off-target effects should not be excluded [12].

On the other hand, henceforth our work prompts the study of those signaling pathways that are overlooked a priori, because ineffective for forward signaling. Our analysis opens the perspective that such systems possibly hide some usefulness in controlling pathways, due to their qualities of retrosignaling. These properties could prove interesting in the fields of drug design or synthetic biology. Indeed we showed that in signaling cascades, novel functionalities can appear precisely in conditions where the biochemical system seems inoperant for forward signaling.

## Methods

### The mathematical model

The kinetic description of the system illustrated in Fig. 1a is deduced by applying the law of mass action to the following reaction scheme (1≤*i*≤*n*):

10a $$\begin{array}{*{20}l} Y_{i}^{0} + Y_{i-1}^{1} &\underset{{d}_{i}^{0}}{\overset{{a_{i}^{0}}}{\rightleftharpoons}} C_{i}^{0} \overset{k_{i}^{0}}{\rightarrow} Y_{i}^{1} + Y_{i-1}^{1}  \end{array} $$

10b $$\begin{array}{*{20}l} Y_{i}^{1} + E_{i} & \underset{{d}_{i}^{1}}{\overset{{a_{i}^{1}}}{\rightleftharpoons}} C_{i}^{1} \overset{k_{i}^{1}}{\rightarrow} Y_{i}^{0} + E_{i}  \end{array} $$

10c $$\begin{array}{*{20}l} Y_{n}^{1} + D & \underset{{d}_{D}}{{\overset{a_D}{\rightleftharpoons}}} C \end{array} $$

We assume that both the activation reaction (10a) and the inactivation reaction (10b) are enzymatic and irreversible.

We denote *Y* the protein (kinase), *C* the enzyme-substrate complex, and *E* the phosphatase, in each cycle. The lower index *i*=1,…,*n* states the cascade level; while the upper indices 0 and 1 refer to variables parameters involved, respectively, in the activation and deactivation reactions. Notably, at any level *i*, protein *Y*_*i*_ is found in either its inactivated or activated form, denoted by ${Y_{i}^{0}}$ or ${Y_{i}^{1}}$, respectively (the upper index can be interpreted e.g. as the absence “0” or the presence “1” of a phosphate group bound to *Y*_*i*_).

The system of non-linear ordinary differential equations (ODEs) corresponding to the kinetic reactions in (10) is given by (1≤*i*≤*n*):

11a $$\begin{array}{*{20}l} &\frac{d{C_{i}^{0}}}{dt} = {a_{i}^{0}}\,{Y_{i}^{0}}\,Y_{i-1}^{1} - ({d_{i}^{0}}+{k_{i}^{0}})\,{C_{i}^{0}}  \end{array} $$

11b $$\begin{array}{*{20}l} &\frac{d{C_{i}^{1}}}{dt} = {a_{i}^{1}}\,{Y_{i}^{1}}\,E_{i} - ({d_{i}^{1}}+{k_{i}^{1}})\,{C_{i}^{1}}  \end{array} $$

11c $$\begin{array}{*{20}l} &\frac{{dC}_{D}}{dt} = a_{D}\,{Y_{n}^{1}}\,D - d_{D}\,C_{D}  \end{array} $$

11d $$\begin{array}{*{20}l} &\frac{d{Y_{i}^{0}}}{dt} = -{a_{i}^{0}}\,{Y_{i}^{0}}\,Y_{i-1}^{1} + {d_{i}^{0}}\,{C_{i}^{0}} + {k_{i}^{1}}\,{C_{i}^{1}}  \end{array} $$

11e $$\begin{array}{*{20}l} &\frac{d{Y_{i}^{1}}}{dt} = -{a_{i}^{1}}\,{Y_{i}^{1}}\,E_{i} + {d_{i}^{1}}\,{C_{i}^{1}} + {k_{i}^{0}}\,{C_{i}^{0}}  \\ &\quad\quad\quad -a^{0}_{i+1} Y_{i+1}^{0} {Y_{i}^{1}} + (d_{i+1}^{0}+k_{i+1}^{0})\,C_{i+1}^{0}, \end{array} $$

with $a_{n+1}^{0}=a_{D}$, $Y_{n+1}^{0} = D$, $d_{n+1}^{0} = d_{D}$, $k_{n+1}^{0} = 0$, and $C_{n+1}^{0} = C_{D}$.

Steady states are obtained by setting the ODEs to zero and using the conservation laws (1≤*i*≤*n*):

12a $$\begin{array}{*{20}l} &E_{iT} = E_{i} + {C_{i}^{1}}, \quad Y_{0T} = {Y_{0}^{1}} + {C_{1}^{0}}, \quad D_{T} = D + C_{D},  \end{array} $$

12b $$\begin{array}{*{20}l} &Y_{iT} = {Y_{i}^{0}} + {Y_{i}^{1}} + {C_{i}^{0}} + {C_{i}^{1}} + C_{i+1}^{0}, \text{with } C_{n+1}^{0}=C_{D} \,.  \end{array} $$

From the sum of (11a) and (11d), we get ${k_{i}^{0}}\,{C_{i}^{0}} - {k_{i}^{1}}\,{C_{i}^{1}} = 0$. From (11a) we have ${C_{i}^{0}} = \frac {Y_{i-1}^{1} {Y_{i}^{0}}}{{K_{i}^{0}}}$; from (11b) and the first equation of (12a) we obtain ${C_{i}^{1}} = E_{iT} \frac {{Y_{i}^{1}}}{{K_{i}^{1}}+{Y_{i}^{1}}}$, where we have defined the Michaelis-Menten constants ${K_{i}^{j}} = ({d_{i}^{j}}+{k_{i}^{j}})/{a_{i}^{j}}$, for *j*={0,1}. In particular, for *i*=1, using the second conservation law of (12a), we can write ${C_{1}^{0}} = Y_{0T} \frac {{Y_{1}^{0}}}{{K_{1}^{0}}+{Y_{1}^{0}}}$. Also, combining (11c) with the third equation of (12a), we get $C_{D} = D_{T} \frac {{Y_{n}^{1}}}{K_{D} + {Y_{n}^{1}}}$, where we have defined the drug dissociation constant *K*_*D*_=*d*_*D*_/*a*_*D*_.

Hence, we end with the following system of 2*n*+2 steady-state equations (1≤*i*≤*n*):

13 $$ \begin{aligned} &{k_{i}^{0}}\,\frac{Y_{i-1}^{1} {Y_{i}^{0}}}{{K_{i}^{0}}} - {k_{i}^{1}}\,E_{iT} \frac{{Y_{i}^{1}}}{{K_{i}^{1}}+{Y_{i}^{1}}} = 0 \\ &Y_{iT} = {Y_{i}^{0}} + {Y_{i}^{1}} + \frac{Y_{i-1}^{1} {Y_{i}^{0}}}{{K_{i}^{0}}} + E_{iT} \frac{{Y_{i}^{1}}}{{K_{i}^{1}}+{Y_{i}^{1}}} + \frac{{Y_{1}^{1}} Y_{i+1}^{0}}{K_{i+1}^{0}} \\ &Y_{0T} = {Y_{0}^{1}} + Y_{0T} \frac{{Y_{1}^{0}}}{{K_{1}^{0}}+{Y_{1}^{0}}}\\ &C_{D}(K_{D}+{Y_{n}^{1}}) - D_{T} \,{Y_{n}^{1}} = 0 \,. \end{aligned}  $$

In particular, we work out the second Eq. 13 with the aim of making it dependent on only one variable, e.g. ${Y_{i}^{1}}$. We replace in the order: ${Y_{i}^{0}} = \frac {{K_{i}^{0}}}{Y_{i-1}^{1}}\,{C_{i}^{0}}$, ${C_{i}^{0}} = \frac {{k_{i}^{1}}}{{k_{i}^{0}}}\,{C_{i}^{1}}$, and ${C_{i}^{1}} = E_{iT} \frac {{Y_{i}^{1}}}{{K_{i}^{1}}+{Y_{i}^{1}}}$. Then we divide by the total protein *Y*_*iT*_ to get

$${}\begin{aligned} 1 & = \frac{{K_{i}^{0}}}{Y_{i-1}^{1}} \frac{{k_{i}^{1}}}{{k_{i}^{0}}} \frac{E_{iT}}{Y_{iT}} \frac{{Y_{i}^{1}}}{{K_{i}^{1}}+{Y_{i}^{1}}} + \left(\frac{{k_{i}^{1}}}{{k_{i}^{0}}} +1\right) \frac{E_{iT}}{Y_{iT}} \frac{{Y_{i}^{1}}}{{K_{i}^{1}}+{Y_{i}^{1}}}\\ & \quad + \frac{{Y_{i}^{1}}}{Y_{iT}} + \frac{k_{i+1}^{1}}{k_{i+1}^{0}} \frac{E_{i+1,T}}{Y_{iT}} \frac{Y_{i+1}^{1}}{K_{i+1}^{1}+Y_{i+1}^{1}} \,. \end{aligned} $$

We finally recover the dimensionless variables $x_{i} = {Y_{i}^{1}}/Y_{iT}$ (*i.e.* the normalized active proteins), and introduce the dimensionless parameters *a*_*i*_, *b*_*i*_, *c*_*i*_, and *e*_*i*_ defined by Eq. (2). Hence, it follows

14 $$ {}\begin{aligned} x_{i-1} = \frac{b_{i} e_{i} x_{i}}{(x_{i}+a_{i})\left(1 \,-\, x_{i} \,-\, e_{i+1} \frac{x_{i+1}}{x_{i+1}+a_{i+1}}\right) - c_{i} x_{i}}, 1\leq i\leq n, \end{aligned}  $$

such that:

15 $$\begin{array}{*{20}l} &x_{0} = 1 - \frac{x_{1}}{s(x_{1}+a_{1})}, \text{with } s = \frac{{k_{1}^{0}} \, Y_{0T}}{{k_{1}^{1}} \, E_{1T}},  \\ &x_{n+1}=x_{n}, \quad a_{n+1}=a_{D}, \quad e_{n+1}=d_{T}.  \end{array} $$

These latter equalities come from the particular cases of *i*=1 and *i*=*n*, to obtain which we replace, respectively, ${C_{1}^{0}} = Y_{0T} \frac {{Y_{1}^{0}}}{{K_{1}^{0}}+{Y_{1}^{0}}}$ and $C_{D} = D_{T} \frac {{Y_{n}^{1}}}{K_{D}+{Y_{n}^{1}}}$, into the second equation of system (13). Explicitly, we can express Eq. 13 as system (1), then more compactly as (3).

### Calculation of the first derivative of *x*_*n*_(*s*)

In the two following sections, we derive the general expressions of slope and curvature of the dose-response function for arbitrary biochemical parameters and cascade length. Let us consider the following general system defined by recursion:

16 $$ \mathbf{z}_{i-1} = \mathbf{F}_{i}(\mathbf{z}_{i})\,,  $$

where $ \mathbf {z}_{i} = \left (\begin {array}{ll} x_{i} \\ y_{i} \end {array} \right)$ and $\mathbf {F}_{i} = \left (\begin {array}{ll} f_{i} \\ g_{i} \end {array}\right)$, for 1≤*i*≤*n*.

By iterative application, we can write **z**_0_ as a function of **z**_*n*_, *i.e.***z**_0_=**F**_1_∘**F**_2_∘…∘**F**_*n*_(**z**_*n*_), and in particular calculate its first derivative with respect to variable *x*_*n*_, according to the chain rule:

17 $$ \mathbf{z}'_{0} = \prod\limits_{1\le i\le n} J_{i}(\mathbf{z}_{i})\cdot \mathbf{z}'_{n} \,,  $$

where $\mathbf {z}'_{i} = \frac {d \mathbf {z}_{i}}{d x_{n}}$, and $J_{i} =\left (\begin {array}{ll} \frac {\partial f_{i}}{\partial x_{i}} \quad \frac {\partial f_{i}}{\partial y_{i}} \\ \frac {\partial g_{i}}{\partial x_{i}} \quad \frac {\partial g_{i}}{\partial y_{i}} \end {array}\right)$ is the jacobian matrix associated to the general dynamical system (16).

Let us now suppose that for 1<*i*≤*n*, $ f_{i}: \mathbf {z}_{i} \mapsto \frac {b_{i} e_{i} x_{i}}{(x_{i}+a_{i})\left (1-x_{i}- e_{i+1} \frac {y_{i}}{y_{i}+a_{i+1}}\right) - c_{i} x_{i}}$,

and *g*_*i*_:**z**_*i*_↦*x*_*i*_ (1≤*i*≤*n*) and set $\quad \mathbf {z}_{0} = \left (\begin {array}{c} s \\ x_{1} \end {array}\right) $.

Thus, $J_{1} =\left (\begin {array}{ll} \frac {\partial \hat {f}_{1}}{\partial x_{1}} \quad \frac {\partial \hat {f}_{1}}{\partial y_{1}}\\ 1 \qquad 0 \end {array}\right) $ (cf. Eq. 3) and $J_{i} =\left (\begin {array}{ll} \frac {\partial f_{i}}{\partial x_{i}} \quad \frac {\partial f_{i}}{\partial y_{i}} \\ 1 \qquad 0 \end {array}\right) $, for 1<*i*≤*n* (assuming *e*_*n*+1_=*d*_*T*_=0, so $\hat f_{n}$ coincides with *f*_*n*_).

It follows that the first component of **z**0′, *i.e.*$s'(x_{n}) = \frac {d s}{d x_{n}}$, is given by

18 $$  s'(x_{n}) = (1 \quad 0)\cdot \prod\limits_{1\le i\le n} J_{i}(x_{i},x_{i+1})\cdot \left(\begin{array}{c} 1 \\ 0 \end{array}\right) \,.  $$

The slope of the dose-response function *x*_*n*_(*s*) at the origin is simply obtained by inverting expression (18) over the biologically relevant domain [0,*α*), evaluating it in *s*=0. Hence, we get

19 $$  {}\begin{aligned} x'_{n}(0) & = \frac{a_{1}}{1+b_{1}} \prod\limits_{2\leq i\leq n} \frac{a_{i}}{b_{i} \,e_{i}} \\ &= \frac{{K_{1}^{1}}}{Y_{1T}+{K_{1}^{0}}} \prod\limits_{2\leq i\leq n} \frac{{k_{i}^{0}} \, Y_{i-1,T} \,{K_{i}^{1}}}{{k_{i}^{1}} \,E_{iT}\,{K_{i}^{0}}} \,. \end{aligned}  $$

Let us note that this formula is derived for an arbitrary cascade with inhomogeneous parameters, describing the contribution of any system’s parameter to the slope of the dose-response function. In particular, for homogeneous parameters, the initial slope is given by Eq. 8.

### Calculation of the second derivative of *x*_*n*_(*s*)

Let us assume that **z**_0_(*x*_*n*_) is a twice differentiable function and let *J*_*i*_ be the jacobian matrix and $ {Hf}_{i} =\left (\begin {array}{ll} \frac {\partial ^{2} f_{i}}{\partial {x_{i}^{2}}} \quad \frac {\partial ^{2} f_{i}}{\partial x_{i}\partial y_{i}} \\ \frac {\partial ^{2} f_{i}}{\partial x_{i}\partial y_{i}} \quad \frac {\partial ^{2} f_{i}}{\partial {y_{i}^{2}}} \end {array}\right) \; \text {and} ~ {Hg}_{i} =\left (\begin {array}{ll} \frac {\partial ^{2} g_{i}}{\partial {x_{i}^{2}}} \quad \frac {\partial ^{2} g_{i}}{\partial x_{i}\partial y_{i}} \\ \frac {\partial ^{2} g_{i}}{\partial x_{i}\partial y_{i}} \quad \frac {\partial ^{2} g_{i}}{\partial {y_{i}^{2}}} \end {array}\right) $ the hessian matrices associated to (16).

Deriving expression (17) with respect to *x*_*n*_ once again, we find

$${{}\begin{aligned} \mathbf{z}^{\prime\prime}_{0} & = \prod\limits_{1\le i\le n} J_{i}(\mathbf{z}_{i}) \cdot \mathbf{z}^{\prime\prime}_{n} + \sum\limits_{0 \leq j < n} \, \prod\limits_{0\leq i \leq j} J_{i}(\mathbf{z}_{i}) \\ & \qquad \cdot \left(\begin{array}{ll} \left(\prod\limits_{k=j+2}^{n} J_{k}(\mathbf{z}_{k}) \cdot \mathbf{z}'_{n} \right)^{T} \cdot {Hf}_{j+1}(\mathbf{z}_{j+1}) \cdot \prod\limits_{k=j+2}^{n} J_{k}(\mathbf{z}_{k}) \cdot \mathbf{z}'_{n} \\ \left(\prod\limits_{k=j+2}^{n} J_{k}(\mathbf{z}_{k}) \cdot \mathbf{z}'_{n} \right)^{T} \cdot {Hg}_{j+1}(\mathbf{z}_{j+1}) \cdot \prod\limits_{k=j+2}^{n} J_{k}(\mathbf{z}_{k}) \cdot \mathbf{z}'_{n} \end{array} \right) \end{aligned}} $$

with *J*_0_=*I*_2_ (the identity matrix 2 × 2).

By selecting the first component of **z**0″, denoted *s*^″^(*x*_*n*_), we calculate the inverse function through the relation

$${x}^{\prime\prime}_{n}(s) = - \frac{{s}^{\prime\prime}(x_{n})}{(s'(x_{n}))^{3}} ~. $$

Although the evaluation of *xn*″(*s*) at the origin makes the expression simpler, the general formula for arbitrary *n* still remains cumbersome, and symbolic computations (e.g. with Maple™) are necessary. As an example, for *n*=3 and homogeneous parameters, the initial curvature is given in Eq. 9.

We remark that, with respect to the initial slope *xn*″(0) depends on the whole parameter set *a*_*i*_,*b*_*i*_,*c*_*i*_, and *e*_*i*_.

### Proof of the lower bound

We demonstrate here that, for homogeneous cascades of arbitrary length *n*, the value of the maximum response *α* for large stimulus *s* is lower bounded by the strictly positive fixed point *x*^∗^ of the equation *x*_*i*−1_=*f*(*x*_*i*_,*x*_*i*+1_), whenever it exists, with *f* being defined in (1b).

We firstly rewrite system (14) as

$$\begin{array}{l} 1 = f(x_{1},x_{2}) \\ x_{i-1} = f(x_{i},x_{i+1}), \quad 1<i<n \\ x_{n-1} = f(x_{n},0) \end{array} $$

where we have considered *s*→+*∞* (implying *x*_0_ tending to 1 from (15)) and *d*_*T*_ fixed to 0 (without loss of generality).

Let us suppose the claim is false, that is *x*_*n*_<*x*^∗^.

By considering the partial derivatives of *f*(*x*,*y*), one can prove that *f* is increasing in *y* for all *x*, and increasing in *x* for *y*=0 or *y*=*x*^∗^.

In fact, from (1b) one calculates $\frac {\partial f}{\partial y} = \frac {a b e x (x+a)}{(y+a)^{2} \left ((x+a) (1-x-ey/(y+a)) - cx \right)^{2}}$ which is always positive, and $\frac {\partial f}{\partial x} = \frac {b e \left (a (1- ey/(y+a)) + x^{2} \right)}{\left ((x+a) (1-x-ey/(y+a)) - cx \right)^{2}}$ which is positive if and only if *a*(1−*ey*/(*y*+*a*))+*x*^2^>0. For *y*=0 the proof is immediate. For *y*=*x*^∗^, we consider the fixed point equation $x^{*}=\frac {b e x^{*}}{(x^{*}+a)(1-x^{*}-e x^{*}/(x^{*}+a)) -c x^{*}}>0$ which implies that the denominator must be positive. Thus, in particular 1−*x*^∗^−*ex*^∗^/(*x*^∗^+*a*)>0 is sufficient and necessary for the positiveness of $\frac {\partial f}{\partial x}(x,x^{*})$.

Hence, we obtain *f*(*x*_*n*_,0)<*f*(*x*_*n*_,*x*^∗^)<*f*(*x*^∗^,*x*^∗^), namely *x*_*n*−1_<*x*^∗^. Then, *f*(*x*_*n*−1_,*x*_*n*_)<*f*(*x*_*n*−1_,*x*^∗^)<*f*(*x*^∗^,*x*^∗^), *i.e.x*_*n*−2_<*x*^∗^. Eventually it follows 1=*x*_0_<*x*^∗^. However, from (7) one can verify that *x*^∗^≤1. Therefore, our claim *x*^∗^≤*x*_*n*_ must be true. □

### The forward signaling regime with homogeneous parameters

In this section, we derive the parameter ranges optimizing the dose-response of a homogeneous cascade, as summarized in Table 1 in Results.

As shown in Fig. 3, we work on an analytical piecewise approximation of the dose-response curve (*cf.* (4) and (5)) based on the initial slope *σ*, the initial curvature *χ*, and the asymptotic value *α* which we proved to be lower bounded by the fixed point *x*^∗^ of function *f*_*n*−1_ (*cf.* system (3)).

We analyze the case for *n*=3. In general, we remark that the forward signaling is enhanced for *d*_*T*_=0, namely when no fraction of the last active protein gets sequestrated by the drug.

We firstly study the sign of the initial curvature *χ*, according to the criteria of efficient forward signaling introduced in Results. From (9) we see that the sign is controlled by the terms *a*+*b* (*a*+*c*−1)−1, *a*+*b* (*a*+*c*−1) and *a*+*c*−1.

In particular, *χ* results to be non-positive if *a*>1. Furthermore, for the criterion above mentioned, the slope and the fixed point have to be as large as possible. Thus, from (8) we require *a*≫*be* and *b*<1, and then *x*^∗^ in (7) is maximized if it also yields *e*≪1 and *c*≪1 (from which it follows *E*_*T*_/*Y*_*T*_≪1, *cf.* Table 1).

Conversely, the positiveness of *χ* is assured by the condition *a*+*b* (*a*+*c*−1)<0 (*i.e.a*/*b*+*a*+*c*−1<0 implying *a*+*b* (*a*+*c*−1)−1<0 and *a*+*c*−1<0 too), which is actually satisfied if *a*≪*b* and *a*,*c*≪1. Moreover, *χ* has to be maximized through the term $\frac {a}{1+b} \left (\frac {a}{be} \right)^{4}$ in Eq. 9, namely if *a*≫*be* and *b*<1. Eventually, all these conditions ensure *x*^∗^ to be already maximized.

### Random sampling of parameters

Although we could achieve some analytical results in the previous sections, these latter are mainly concerned with the regimes of forward signaling (*jk*1), *j*,*k*∈{0,1} in cascades with homogeneous parameters. To go further we perform numerical investigations by randomly sampling the full parameter space, and then classify statistically all the parameter sets according to some characteristics of the response functions they give rise to. The objective is to point out the typical values of parameter that favor one of the 7 signaling regimes (*jkl*)≠(000). The main tool which is considered below is to seek for parameter conditions that maximize the so-called likelihood of the parameters in the various regimes (*cf.* Eq. (21) below).

As we want to look for conditions on parameters that can be formulated in terms of dimensionless parameters, we consider ratios of biochemical parameters to be analyzed. On the other hand we first sample the following 20 biochemical parameters: total phosphatases *E*_*iT*_, total kinases *Y*_*iT*_, Michaelis-Menten constants ${k_{i}^{0}}$, ${k_{i}^{1}}$, catalytic rates ${k_{i}^{0}}$, ${k_{i}^{1}}$. The 20 biochemical parameters were sampled in logarithmic scale, uniformly in the range 10^−2^ to 10^2^ generating 1.000.000 sets, using Latin hypercube method [26, 27]. This technique consists in dividing the hyperspace $\mathbb {R}^{20}$ into 1.000.000 intervals for each parameter. A random set is formed by selecting one random interval for each parameter *without replacement*, and with those values, constructing one set of values for the 20 different biochemical parameters we focused on. This method guarantees an exhaustive exploration of the hyperspace.

As the Michaelis-Menten constants are parameters which condense information in equilibrium for enzymatic cycles but no dynamic rates, sampling this constants means an extra degree of freedom to choose some of the dynamic rates, in this work we choose ${d_{i}^{j}}=1$ and use ${K_{i}^{j}}$ to compute ${a_{i}^{j}}=\frac {{d_{i}^{j}}+{k_{i}^{j}}}{{K_{i}^{j}}}$, for 1≤*i*≤*n*, *j*∈{0,1}. Finally, with all the dynamic rates, we solved the ODEs for two different scenarios:

1. *Stimulus*→+*∞*, *Drug*=0
2. *Stimulus*→+*∞*, *Drug*→+*∞*

To take into account the condition *Stimulus*→+*∞* we replaced, for the first cycle, Eq. (11d) by $\frac {d {Y_{1}^{0}}}{dt}=0$ and Eq. 11a by $\frac {d {C_{1}^{0}}}{dt}={C_{1}^{1}}{k_{1}^{1}}-{C_{1}^{0}}{k_{0}^{1}}$ (see Additional file 6).

Those replacements amount to remove the inactive protein in cycle 1 because, when *Stimulus*→+*∞*, this protein is either in its active state or in the complexes ${C_{1}^{j}}$. About the second scenario, if *Drug*→+*∞* the protein in cycle 3 is all bound to the drug. Therefore from (11), we remove the equations for cycle 3 and set ${Y_{3}^{j}}=0$ on the equation for the second cycle that is coupled to ${Y_{3}^{j}}$.

With all this information, for each set of random parameters we solved the ODEs for the two scenarios, the initial condition of the system being:

$$\begin{array}{l} {Y_{1}^{0}} = 0, \quad {C^{0}_{1}}= Y_{1T}/2, \quad {C^{1}_{1}}= Y_{1T}/2 \\ {Y_{2}^{0}} = Y_{2T} \\ {Y_{3}^{0}} = Y_{3T} \\ \end{array} $$

We remark that for infinite stimulus, there is no ${Y_{1}^{0}}$ and then the initial condition is equally distributed in the intermediate complex ${C_{1}^{j}}$.

After we solved the ODEs, we computed the following dimensionless output variables:

$$\begin{array}{@{}rcl@{}} \bigtriangleup x_{1} &=& x_{1}(d_{T} \rightarrow +\infty,s \rightarrow +\infty)-x_{1}(d_{T}=0, s \rightarrow +\infty),\\ \bigtriangleup x_{2} &=& x_{2}(d_{T} \rightarrow +\infty, s \rightarrow +\infty)-x_{2}(d_{T}=0, s \rightarrow +\infty),\\ \bigtriangleup x_{3} &=& x_{3}(d_{T}=0, s \rightarrow +\infty)-x_{3}(d_{T}=0,s=0). \end{array} $$

These three dimensionless outputs are the second order retroactivity, first order retroactivity, and forward activity, respectively. The numerical simulation also computes slope, curvature, and fixed point using (19), (9), and (7), respectively.

#### The likelihood of parameters for each signaling regime

In this study we consider two types of parameters, either dimensional or dimensionless. The dimensional set involves total concentrations of kinases and phosphatases, Michaelis-Menten constants and catalytic rates for the enzymatic reactions. The dimensionless set involves combinations of parameters that appear when solving the dynamical equations for the steady-states, and also ratios of parameters with the same units (ratios of rates, ratios of concentrations, etc).

For each dimensionless parameters (denoted generically by *λ*) and for each regime (*jkl*), we construct the following probability using Bayes theorem:

20 $$ P(jkl | \lambda) = \frac{P(\lambda | jkl) P(jkl)}{P(\lambda)}  $$

with *P*(*jkl*|*λ*) being the probability of finding the signaling regime (*jkl*), given some specific parameter value *λ*, *P*(*λ*|*jkl*) the probability distribution of parameter *λ* given a specific signaling regime, *P*(*jkl*) the probability to obtain each regime and *P*(*λ*) the probability distribution of parameter *λ*, whatever the regime. The last one is obtained analytically as a sum of 2 (or 4) uniform distributions.

Let us note that when (*jkl*) is fixed, the function *P*(*jkl*|*λ*) is not a probability distribution over *λ*, but is called the *likelihood* of *λ*, for a given regime (*jkl*), see [28]. One main goal of our numerical simulations is to draw and to compare the curves of normalized likelihoods, defined by:

21 $$ L_{jkl} (\lambda) = \frac{P(jkl | \lambda) }{\max_{\lambda} P(jkl | \lambda)}  $$

The reason why we normalize by the the maximum is that the maximum probability of each regimes can be quite different (see Table 1 in Additional file 1, and Fig. 1b where the probability of each regime *P*(*jkl*) is shown).

Therefore a rational criterion to characterize parameter values that promote a given signaling regime (*jkl*) is to look for values which maximize the corresponding likelihood function.

Then, considering signaling cascades with inhomogeneous parameters, simulations of the dose-response curves and of the drug-response curves were done for 1.000.000 random sets of biochemical parameters. The histograms of parameters were classified according to the 8 different regimes. Then, using Eq. 20, the likelihood functions of the considered dimensionless parameters were drawn on separate graphs for all possible regimes (Fig. 4 and Additional file 2).

Let us remark that, despite the large number of samplings, we observe that some signaling regimes are rare. Therefore we computed error bars for all the likelihood curves. For each specific signaling regime the error bars were constructed on the parameter histogram by using a binomial probability *p* of being in the *i*-th bin and 1−*p* of being in another bin. Then using error propagation formula for Bayes relation (20) we obtained the error of each parameter curve.

## Additional files

Additional file 1 The inverse iterative map. (PDF 88.9 kb)

Additional file 2 Likelihood curves and maxima. (PDF 483 kb)

Additional file 3 Graphical representation of the signaling regimes. (PDF 1290 kb)

Additional file 4 Threshold robustness. (PDF 249 kb)

Additional file 5 Table of probabilities of signaling regimes. (PDF 56.5 kb)

Additional file 6 Infinite drug and infinite stimulus limits. (PDF 77.9 kb)

## Abbreviations

LHS: Latin hypercube sampling
MAPK: Mitogen-activated protein kinase
ODE: Ordinary differential equation
