# Supplementary material for: Signaling cascades transmit information downstream and upstream but unlikely simultaneously
Source: BMC Syst Biol. 2016 Aug 25;10(1):84. doi: 10.1186/s12918-016-0303-2 (PMC5000522; doi:10.1186/s12918-016-0303-2)
Supplement: Additional file 1 — The inverse iterative map. (PDF 88.9 kb) [file 12918_2016_303_MOESM1_ESM.pdf]

Additional File 1

The inverse iterative map

The inverse map:

$$
\begin{aligned}
x_2 &= \check{g}_1(s, x_1) \\
x_{i+1} &= g_i(x_{i-1}, x_i), \qquad 1 \leq i \leq n \\
d_T &= \hat{g}_n(x_n, x_{n-1}).
\end{aligned}
$$

Explicitly we have:

$$
\begin{aligned}
x_2 &= \frac{a_2 \left\{ \left(s - \frac{x_1}{x_1 + a_1} \right) ((x_1 + a_1)(1 - x_1) - c_1 x_1) - b_1 x_1 \right\}}{\left(s - \frac{x_1}{x_1 + a_1} \right) ((x_1 + a_1)(-1 + x_1 + e_2) + c_1 x_1) + b_1 x_1}, \\
x_{i+1} &= \frac{a_{i+1} \left\{ x_{i-1} \left((x_i + a_i)(1 - x_i) - c_i x_i \right) - b_i e_i x_i \right\}}{x_{i-1}((x_i + a_i)(-1 + x_i + e_{i+1}) + c_i x_i) + b_i e_i x_i}, \quad 1 < i < n, \\
d_T &= \frac{(x_n + a_D) \left\{ x_{n-1} \left((x_n + a_n)(1 - x_n) - c_n x_n \right) - b_n e_n x_n \right\}}{x_{n-1} x_n (x_n + a_n)}.
\end{aligned}
$$

By setting $x_0 = 1 - \frac{x_1}{s(x_1 + a_1)}$, we can write $\check{g}_1(s, x_1) = g_1(x_0, x_1)$.
