# Supplementary material for: Signaling cascades transmit information downstream and upstream but unlikely simultaneously
Source: BMC Syst Biol. 2016 Aug 25;10(1):84. doi: 10.1186/s12918-016-0303-2 (PMC5000522; doi:10.1186/s12918-016-0303-2)
Supplement: Additional file 2 — Likelihood curves and maxima. (PDF 483 kb) [file 12918_2016_303_MOESM2_ESM.pdf]

Additional File 2

Normalized likelihoods curves

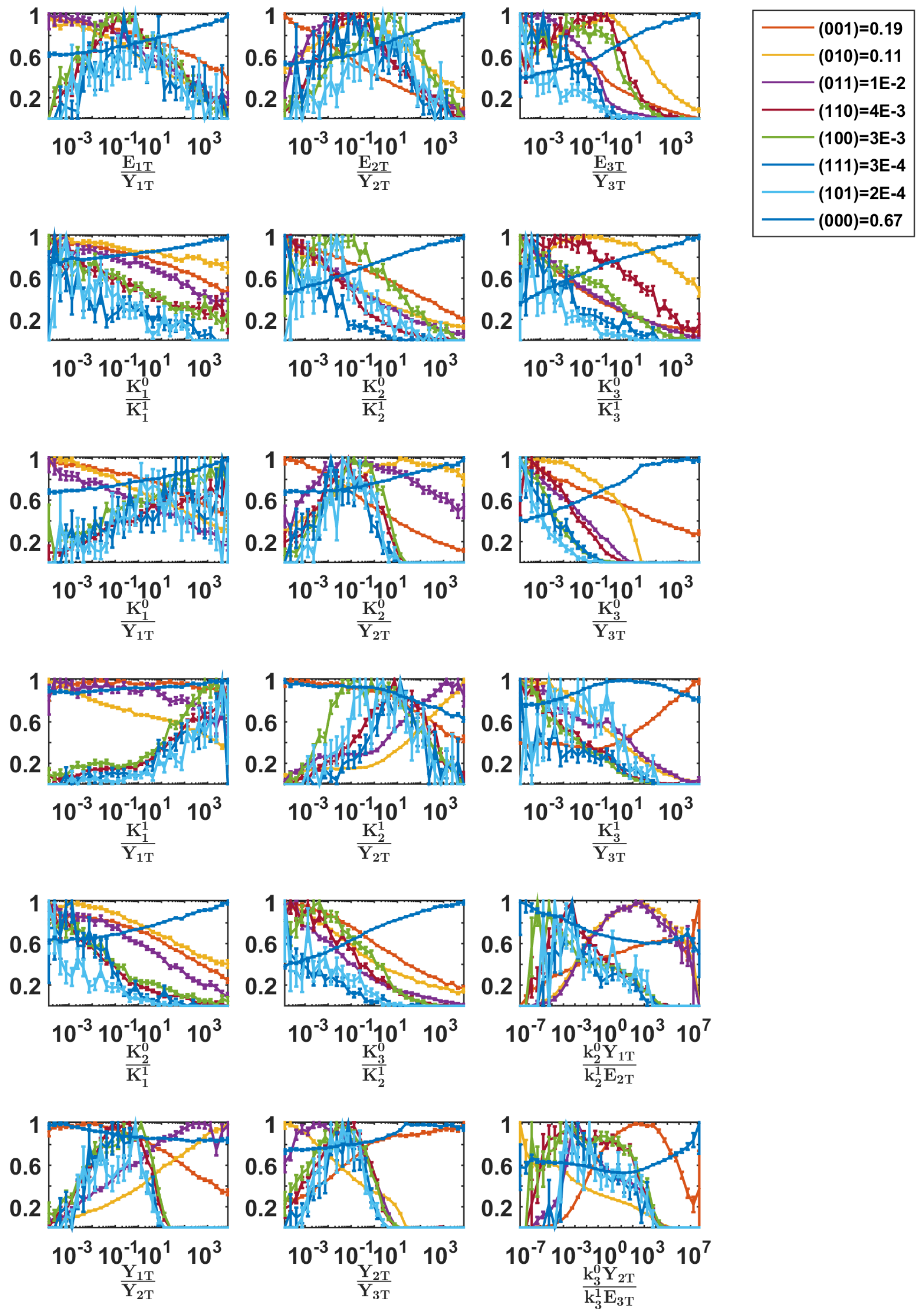

Figure 1: **Likelihood curves normalized by their maximum.**

Tables of likelihood maxima

The likelihood curves are normalized by each maximum, the maximum value of each likelihood curve is in Tables 1 and 2.

	(000)	(001)	(010)	(011)	(100)	(101)	(110)	(111)
$\frac{k_2^0 Y_{1T}}{k_2^1 E_{2T}}$	0,981	0,394	0,149	0,024	0,011	0,001	0,011	0,001
$\frac{k_3^0 Y_{2T}}{k_3^1 E_{3T}}$	1,182	0,266	0,392	0,033	0,005	0,000	0,007	0,001
$\frac{K_2^0}{K_1^1}$	0,874	0,252	0,147	0,030	0,010	0,002	0,016	0,001
$\frac{K_3^0}{K_2^1}$	0,912	0,312	0,240	0,057	0,008	0,001	0,013	0,002
$\frac{Y_{1T}}{Y_{2T}}$	0,756	0,232	0,262	0,028	0,007	0,001	0,009	0,001
$\frac{Y_{2T}}{Y_{3T}}$	0,766	0,247	0,323	0,038	0,009	0,001	0,011	0,001
$\frac{K_1^0}{K_1^1}$	0,777	0,235	0,132	0,025	0,007	0,001	0,009	0,001
$\frac{K_2^0}{K_2^1}$	0,889	0,287	0,232	0,042	0,006	0,001	0,015	0,002
$\frac{K_3^0}{K_3^1}$	0,895	0,500	0,126	0,046	0,008	0,002	0,007	0,001
$\frac{E_{1T}}{Y_{1T}}$	0,866	0,232	0,179	0,026	0,005	0,000	0,007	0,001
$\frac{E_{2T}}{Y_{2T}}$	0,920	0,403	0,144	0,026	0,006	0,000	0,007	0,001
$\frac{E_{3T}}{Y_{3T}}$	0,988	0,472	0,149	0,051	0,007	0,002	0,008	0,001
$\frac{Y_{1T}}{K_1^1}$	0,716	0,196	0,163	0,020	0,011	0,001	0,017	0,001
$\frac{Y_{2T}}{K_2^1}$	0,726	0,223	0,371	0,039	0,005	0,000	0,008	0,001
$\frac{Y_{3T}}{K_3^1}$	0,715	0,408	0,223	0,034	0,015	0,000	0,016	0,001
$\frac{Y_{1T}}{K_1^0}$	0,832	0,232	0,166	0,028	0,006	0,000	0,008	0,001
$\frac{Y_{2T}}{K_2^0}$	0,828	0,350	0,138	0,020	0,007	0,001	0,010	0,001
$\frac{Y_{3T}}{K_3^0}$	0,905	0,296	0,204	0,065	0,033	0,004	0,020	0,003

Table 1: Maxima for inhomogeneous parameters.
