# Supplementary material for: Signaling cascades transmit information downstream and upstream but unlikely simultaneously
Source: BMC Syst Biol. 2016 Aug 25;10(1):84. doi: 10.1186/s12918-016-0303-2 (PMC5000522; doi:10.1186/s12918-016-0303-2)
Supplement: Additional file 3 — Graphical representation of the signaling regimes. (PDF 1290 kb) [file 12918_2016_303_MOESM3_ESM.pdf]

Additional File 3

Graphical representation of the signaling regimes

We detail the procedure to obtain the motifs (Fig.5 in the paper) by means of the analysis of the regimes' likelihood curves based on the algorithm presented in the main paper.

Regime (001)

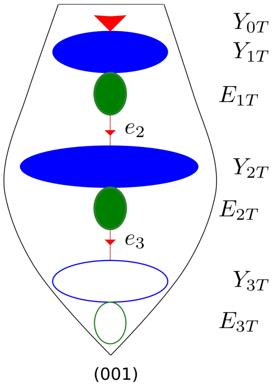

1. Relative sizes of the total proteins:

   $Y_{1T} < Y_{2T}, \quad Y_{2T} > Y_{3T}$

2. Relative sizes of the total phosphatases:

   $E_{1T} \ll Y_{1T}, \quad E_{2T} \ll Y_{2T}, \quad E_{3T} \ll Y_{3T}$

3. Cycle activation/deactivation:

   $1/e_2 \gg 1, \quad 1/e_3 > 1$

4. Saturation/Non-saturation of kinases Y_0^1, Y_1^1, Y_2^1:

   $K_1^0 \ll Y_{1T}, \quad K_2^0 \ll Y_{2T}, \quad K_3^0 \ll Y_{3T}$

5. Saturation/Non-saturation of phosphatases E_1, E_2, E_3:

   $K_1^1 \sim Y_{1T}, \quad K_2^1 \ll Y_{2T}, \quad K_3^1 \gg Y_{3T}$

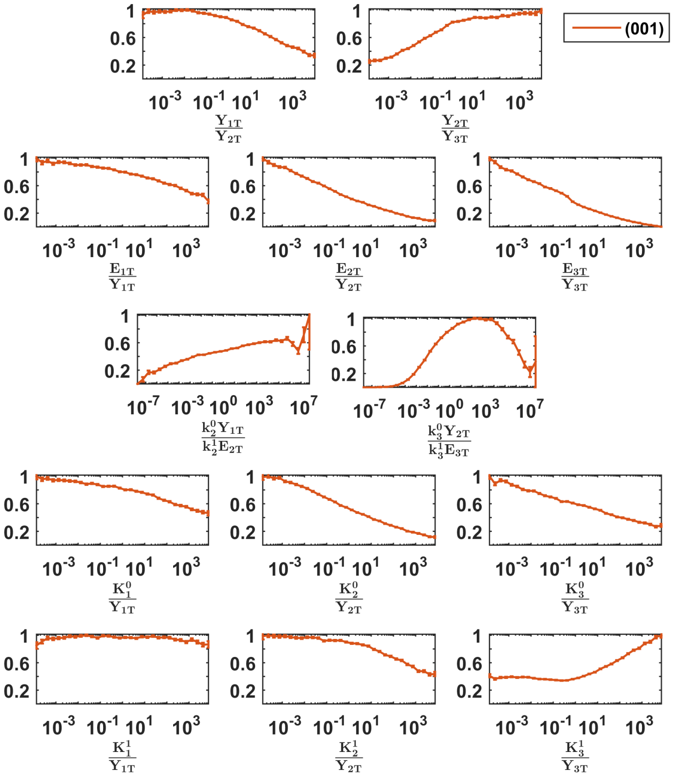

Normalized likelihood curves of regime (001).

Regime (010)

1. Relative sizes of the total proteins:
   $Y_{1T} > Y_{2T}, \quad Y_{2T} < Y_{3T}$
2. Relative sizes of the total phosphatases:
   $E_{1T} \ll Y_{1T}, \quad E_{2T} \sim Y_{2T}, \quad E_{3T} \sim Y_{3T}$
3. Cycle activation/deactivation:
   $1/e_2 > 1, \quad 1/e_3 < 1$
4. Saturation/Non-saturation of kinases Y_0^1, Y_1^1, Y_2^1:
   $K_1^0 \ll Y_{1T}, \quad K_2^0 \geq Y_{2T}, \quad K_3^0 \ll Y_{3T}$
5. Saturation/Non-saturation of phosphatases E_1, E_2, E_3:
   $K_1^1 \ll Y_{1T}, \quad K_2^1 \gg Y_{2T}, \quad K_3^1 \ll Y_{3T}$

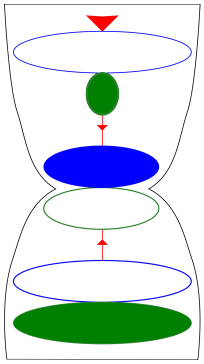

(010)

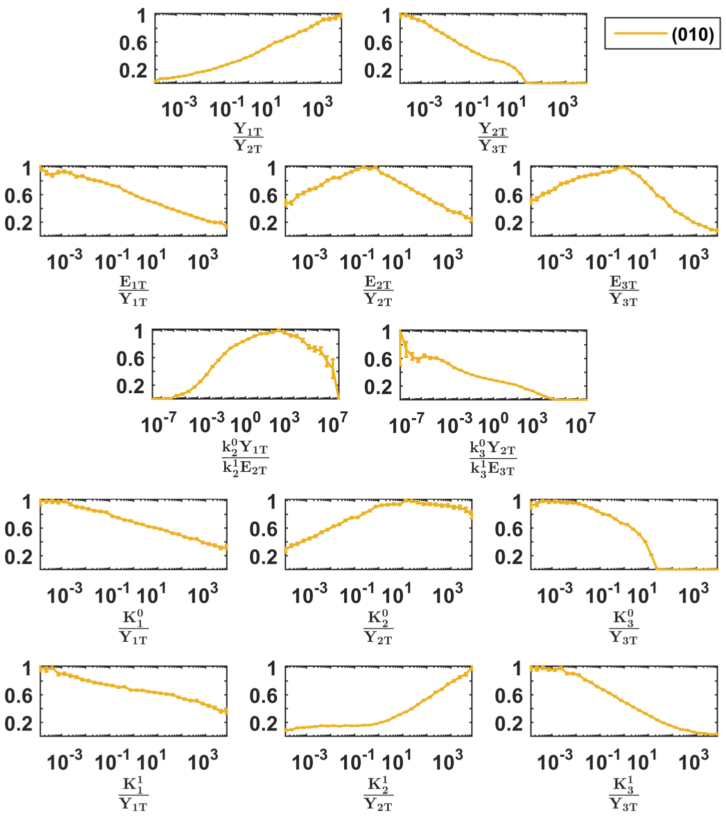

Normalized likelihood curves of regime (010).

Regime (011)

1. Relative sizes of the total proteins:

   $Y_{1T} > Y_{2T}, \quad Y_{2T} < Y_{3T}$

2. Relative sizes of the total phosphatases:

   $E_{1T} \ll Y_{1T}, \quad E_{2T} \sim Y_{2T}, \quad E_{3T} \ll Y_{3T}$

3. Cycle activation/deactivation:

   $1/e_2 > 1, \quad 1/e_3 < 1$

4. Saturation/Non-saturation of kinases Y_0^1, Y_1^1, Y_2^1:

   $K_1^0 \ll Y_{1T}, \quad K_2^0 \sim Y_{2T}, \quad K_3^0 \ll Y_{3T}$

5. Saturation/Non-saturation of phosphatases E_1, E_2, E_3:

   $K_1^1 \ll Y_{1T}, \quad K_2^1 \gg Y_{2T}, \quad K_3^1 \ll Y_{3T}$

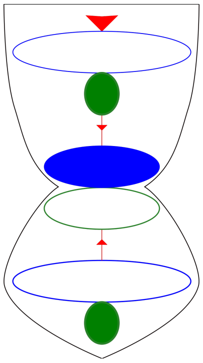

(011)

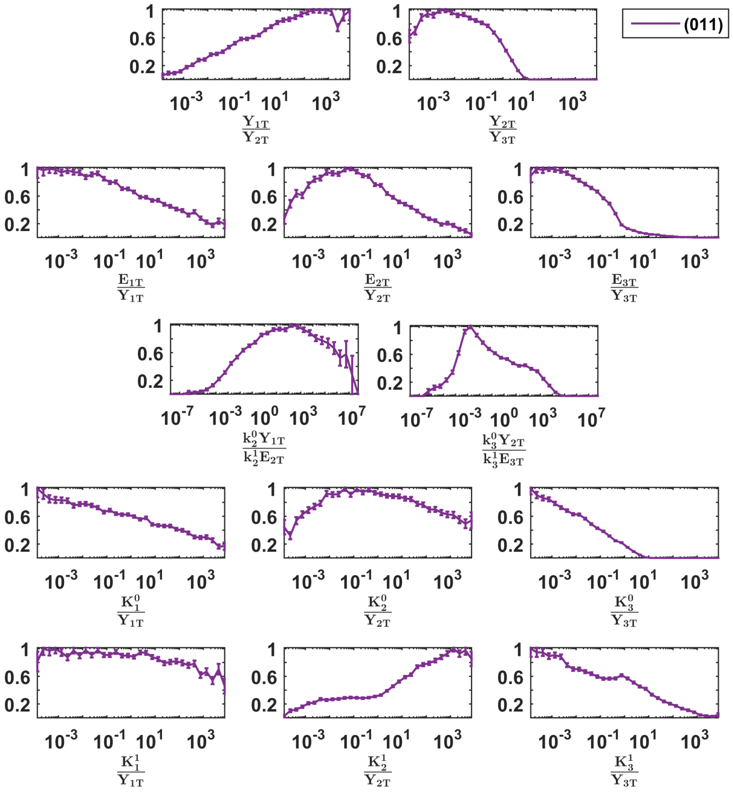

Normalized likelihood curves of regime (011).

Regimes (100) or (110)

1. Relative sizes of the total proteins:

   $Y_{1T} \leq Y_{2T}, \quad Y_{2T} < Y_{3T}$

2. Relative sizes of the total phosphatases:

   $E_{1T} \sim Y_{1T}, \quad E_{2T} \sim Y_{2T}, \quad E_{3T} \sim Y_{3T}$

3. Cycle activation/deactivation:

   $1/e_2 < 1, \quad 1/e_3 < 1$

4. Saturation/Non-saturation of kinases Y_0^1, Y_1^1, Y_2^1:

   $K_1^0 \gg Y_{1T}, \quad K_2^0 < Y_{2T}, \quad K_3^0 \ll Y_{3T}$

5. Saturation/Non-saturation of phosphatases E_1, E_2, E_3:

   $K_1^1 \gg Y_{1T}, \quad K_2^1 \sim Y_{2T}, \quad K_3^1 \ll Y_{3T}$

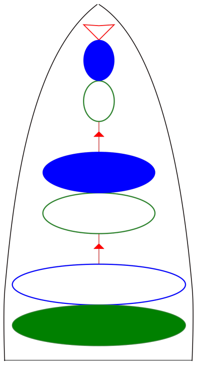

(1k0)

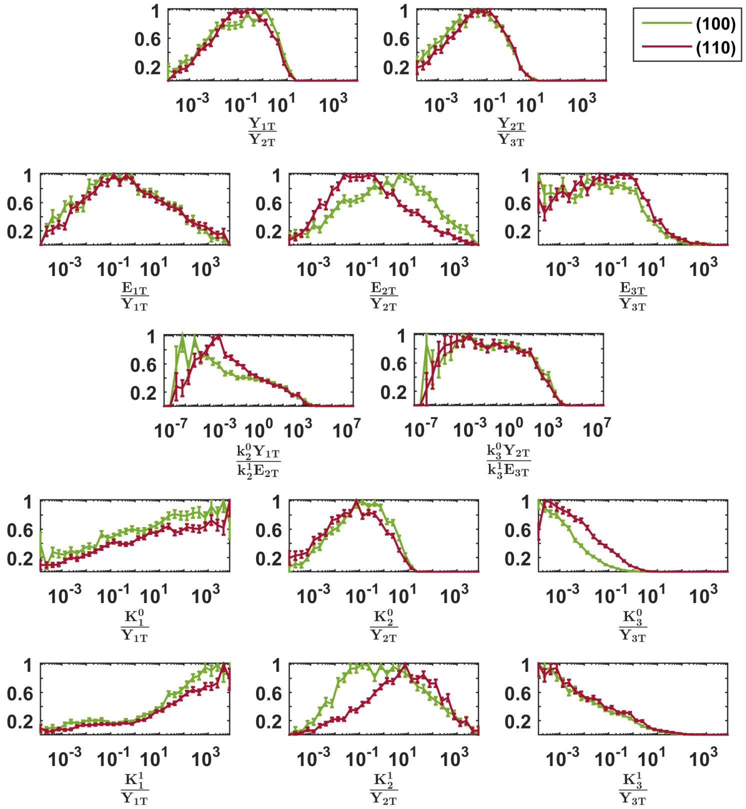

Normalized likelihood curves of regimes (100) and (110).

Regimes (101) or (111)

1. Relative sizes of the total proteins:

   $Y_{1T} \sim Y_{2T}, \quad Y_{2T} < Y_{3T}$

2. Relative sizes of the total phosphatases:

   $E_{1T} \sim Y_{1T}, \quad E_{2T} \sim Y_{2T}, \quad E_{3T} \ll Y_{3T}$

3. Cycle activation/deactivation:

   $1/e_2 < 1, \quad 1/e_3 < 1$

4. Saturation/Non-saturation of kinases Y_0^1, Y_1^1, Y_2^1:

   $K_1^0 > Y_{1T}, \quad K_2^0 < Y_{2T}, \quad K_3^0 \ll Y_{3T}$

5. Saturation/Non-saturation of phosphatases E_1, E_2, E_3:

   $K_1^1 \gg Y_{1T}, \quad K_2^1 \sim Y_{2T}, \quad K_3^1 < Y_{3T}$

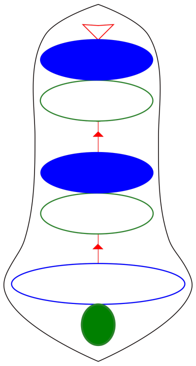

(1k1)

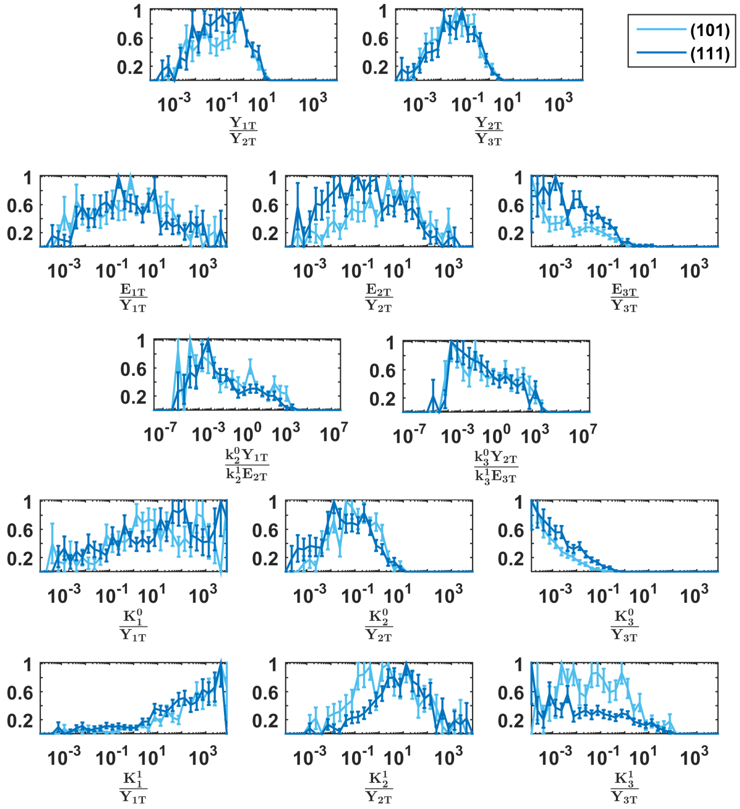

Normalized likelihood curves of regimes (101) and (111).
