# Supplementary material for: Signaling cascades transmit information downstream and upstream but unlikely simultaneously
Source: BMC Syst Biol. 2016 Aug 25;10(1):84. doi: 10.1186/s12918-016-0303-2 (PMC5000522; doi:10.1186/s12918-016-0303-2)
Supplement: Additional file 4 — Threshold robustness. (PDF 249 kb) [file 12918_2016_303_MOESM4_ESM.pdf]

Additional File 4

Threshold robustness

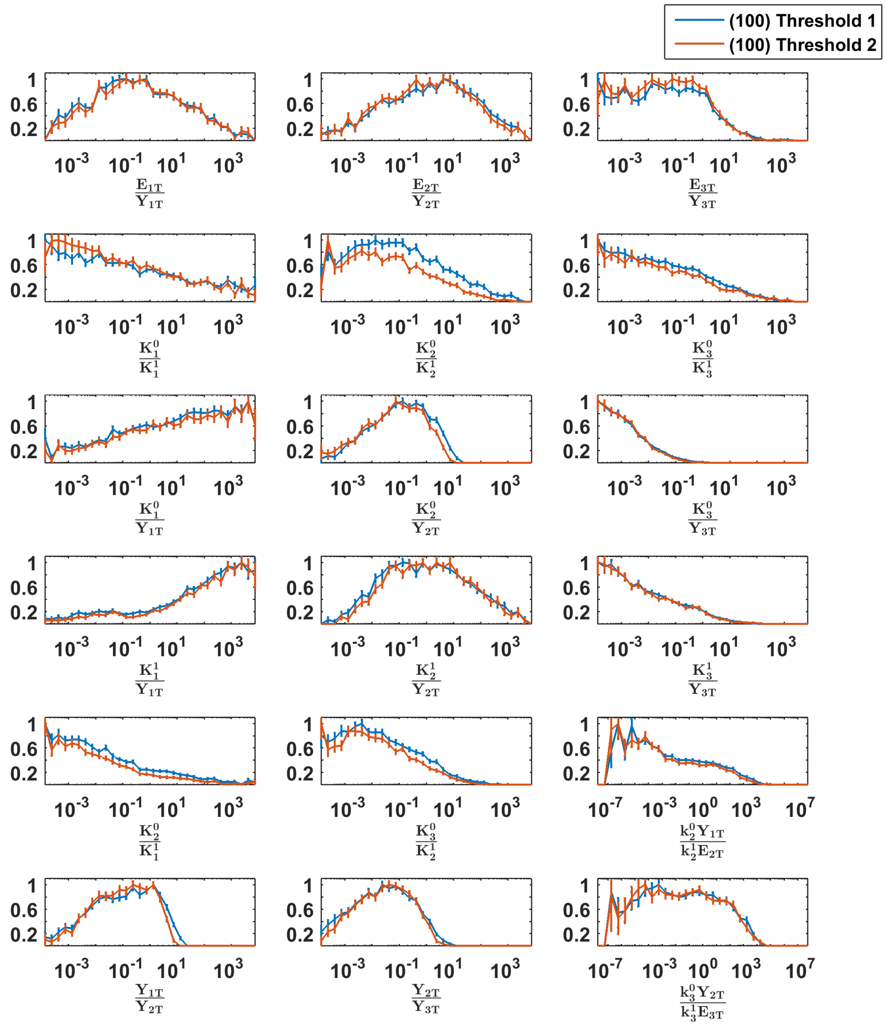

Figure 1: **Likelihood curves for regime (100) normalized by their maximum.** Threshold 1: $\Delta x_1 > 5\%$, $\Delta x_2 > 5\%$, $\Delta x_3 > 50\%$. Threshold 2: $\Delta x_1 > 10\%$, $\Delta x_2 > 10\%$, $\Delta x_3 > 75\%$.
