# Supplementary material for: Signaling cascades transmit information downstream and upstream but unlikely simultaneously
Source: BMC Syst Biol. 2016 Aug 25;10(1):84. doi: 10.1186/s12918-016-0303-2 (PMC5000522; doi:10.1186/s12918-016-0303-2)
Supplement: Additional file 5 — Table of probabilities of signaling regimes. (PDF 56.5 kb) [file 12918_2016_303_MOESM5_ESM.pdf]

Additional File 5

Table of probabilities of signaling regimes

Based on the parameter conditions deduced from the likelihood curves, and illustrated by the motifs (Fig.5 in the paper), the probabilities for the regimes are modified as reported in the following table (*cf.* Fig.6 in the paper).

Parameter Restrictions	Regime Probabilities (in %)				
	(001)	(010)	(011)	(1k0)	(1k1)
none	19.05	11.46	1.77	0.87	0.05
restriction (001)	**94.83**	1.15	3.45	0	0
restriction (010)	3.77	**82.36**	7.71	0	0
restriction (011)	17.85	61.07	**20**	0.36	0.36
restriction (1k0)	6.03	24.62	4.52	**37.94**	1.76
restriction (1k1)	22.45	17.69	7.14	28.23	**3.74**

Table 1: The restrictions on parameter ranges (corresponding to the 5 different motifs) actually increase the probability of the considered (possibly hybrid) regime.
