# Supplementary material for: Signaling cascades transmit information downstream and upstream but unlikely simultaneously
Source: BMC Syst Biol. 2016 Aug 25;10(1):84. doi: 10.1186/s12918-016-0303-2 (PMC5000522; doi:10.1186/s12918-016-0303-2)
Supplement: Additional file 6 — Infinite drug and infinite stimulus limits. (PDF 77.9 kb) [file 12918_2016_303_MOESM6_ESM.pdf]

Additional File 6

Infinite Drug, Infinite Stimulus limits

In order to keep accuracy on the results of the dynamic simulations, we took analytically the limits of infinite drug and stimulus instead of using a high value to simulate it. Given the ODEs of the three cycle cascade, the infinite stimulus limit would affect the first protein: it must be in its active state or sequestrated in one of the two intermediate complexes, but it can't be inactive because we assume immediate activation. Infinite drug would imply no unbounded state for the third kinase, being completly sequestrated by the drug.

Given explicitly the set of the ODEs for the three-cycle cascade,

$$\frac{dY_1^1}{dt} = C_1^0 k_1^0 + C_1^1 d_1^1 + C_2^0(d_2^0 + k_2^0) - Y_1^1(E_1 a_1^1 - Y_2^0 a_2^0) \tag{1a}$$

$$\frac{dY_1^0}{dt} = C_1^0 d_1^0 + C_1^1 k_1^1 - Y_0^1 Y_1^0 a_1^0 \tag{1b}$$

$$\frac{dC_1^0}{dt} = Y_0^1 Y_1^0 a_1^0 - C_1^0(d_1^0 + k_1^0) \tag{1c}$$

$$\frac{dC_1^1}{dt} = E_1 Y_1^1 a_1^1 - C_1^1(d_1^1 + k_1^1) \tag{1d}$$

$$\frac{dY_2^1}{dt} = C_2^0 k_2^0 + C_2^1 d_2^1 + C_3^0(d_3^0 + k_3^0) - Y_2^1(E_2 a_2^1 - Y_3^0 a_3^0) \tag{1e}$$

$$\frac{dY_2^0}{dt} = C_2^0 d_2^0 + C_2^1 k_2^1 - Y_1^1 Y_2^0 a_2^0 \tag{1f}$$

$$\frac{dC_2^0}{dt} = Y_1^1 Y_2^0 a_2^0 - C_2^0(d_2^0 + k_2^0) \tag{1g}$$

$$\frac{dC_2^1}{dt} = E_2 Y_2^1 a_2^1 - C_2^1(d_2^1 + k_2^1) \tag{1h}$$

$$\frac{dY_3^1}{dt} = C_3^0 k_3^0 + C_3^1 d_3^1 + d_D C - Y_3^1(D a_D + E_3 a_3^1) \tag{1i}$$

$$\frac{dY_3^0}{dt} = C_3^0 d_3^0 + C_3^1 k_3^1 - Y_2^1 Y_3^0 a_3^0 \tag{1j}$$

$$\frac{dC_3^0}{dt} = Y_2^1 Y_3^0 a_3^0 - C_3^0(d_3^0 + k_3^0) \tag{1k}$$

$$\frac{dC_3^1}{dt} = E_3 Y_3^1 a_3^1 - C_3^1(d_3^1 + k_3^1) \tag{1l}$$

$$\frac{dC}{dt} = Y_3^1 D a_D - C d_D \tag{1m}$$

The infinite stimulus limit modifications are:

$$\frac{dY_1^0}{dt} = 0 \tag{2a}$$

$$\frac{dC_1^0}{dt} = C_1^1 k_1^1 - C_1^0 k_1^0 \tag{2b}$$

While the infinite drug limit modifications:

$$\frac{dY_2^1}{dt} = C_2^0 k_2^0 + C_2^1 d_2^1 - E_2 Y_2^1 a_2^1 \tag{3a}$$

$$\frac{dC_2^1}{dt} = E_2 Y_2^1 a_2^1 - C_2^1 (d_2^1 + k_2^1) \tag{3b}$$

$$\frac{dY_3^1}{dt} = 0 \tag{3c}$$

$$\frac{dY_3^0}{dt} = 0 \tag{3d}$$

$$\frac{dC_3^0}{dt} = 0 \tag{3e}$$

$$\frac{dC_3^1}{dt} = 0 \tag{3f}$$

$$\frac{dC}{dt} = 0 \tag{3g}$$
